# Comparing the Psychometric Properties of Two Physical Activity Self-Efficacy Instruments in Urban, Adolescent Girls: Validity, Measurement Invariance, and Reliability

**DOI:** 10.3389/fpsyg.2017.01301

**Published:** 2017-08-03

**Authors:** Vicki R. Voskuil, Steven J. Pierce, Lorraine B. Robbins

**Affiliations:** ^1^Department of Nursing, Hope College, Holland MI, United States; ^2^Center for Statistical Training and Consulting, Michigan State University, East Lansing MI, United States; ^3^College of Nursing, Michigan State University, East Lansing MI, United States

**Keywords:** measurement invariance, physical activity, self-efficacy, adolescents, girls

## Abstract

**Aims:** This study compared the psychometric properties of two self-efficacy instruments related to physical activity. Factorial validity, cross-group and longitudinal invariance, and composite reliability were examined.

**Methods:** Secondary analysis was conducted on data from a group randomized controlled trial investigating the effect of a 17-week intervention on increasing moderate to vigorous physical activity among 5th–8th grade girls (*N* = 1,012). Participants completed a 6-item Physical Activity Self-Efficacy Scale (PASE) and a 7-item Self-Efficacy for Exercise Behaviors Scale (SEEB) at baseline and post-intervention. Confirmatory factor analyses for intervention and control groups were conducted with Mplus Version 7.4 using robust weighted least squares estimation. Model fit was evaluated with the chi-square index, comparative fit index, and root mean square error of approximation. Composite reliability for latent factors with ordinal indicators was computed from Mplus output using SAS 9.3.

**Results:** Mean age of the girls was 12.2 years (*SD* = 0.96). One-third of the girls were obese. Girls represented a diverse sample with over 50% indicating black race and an additional 19% identifying as mixed or other race. Both instruments demonstrated configural invariance for simultaneous analysis of cross-group and longitudinal invariance based on alternative fit indices. However, simultaneous metric invariance was not met for the PASE or the SEEB instruments. Partial metric invariance for the simultaneous analysis was achieved for the PASE with one factor loading identified as non-invariant. Partial metric invariance was not met for the SEEB. Longitudinal scalar invariance was achieved for both instruments in the control group but not the intervention group. Composite reliability for the PASE ranged from 0.772 to 0.842. Reliability for the SEEB ranged from 0.719 to 0.800 indicating higher reliability for the PASE. Reliability was more stable over time in the control group for both instruments.

**Conclusions:** Results suggest that the intervention influenced how girls responded to indicator items. Neither of the instruments achieved simultaneous metric invariance making it difficult to assess mean differences in PA self-efficacy between groups.

## Introduction

Despite the benefits of physical activity (PA), less than 25% of adolescents meet recommended guidelines (Fakhouri et al., 2014; Kann et al., 2016) calling for 60 min or more per day of at least moderate-intensity PA (U.S. Department of Health and Human Services, 2008). Compared to boys, girls attain less PA and have greater declines in the behavior throughout adolescence (Dumith et al., 2011). Increased understanding of factors underlying this occurrence among adolescent girls is urgently needed, particularly given that most interventions have not increased PA in this population (Camacho-Minano et al., 2011).

While many psychosocial factors have been theorized to increase PA among adolescents, self-efficacy is an important correlate and determinant of PA (Craggs et al., 2011; Bauman et al., 2012) and mediator of PA intervention effects (Lubans et al., 2008). However, some researchers have reported contradictory findings regarding the relationship between self-efficacy and adolescent PA suggesting that inadequate and varied measurement of the concept may explain the inconsistencies (Dewar et al., 2013; Plotnikoff et al., 2013).

Establishing multi-group and longitudinal invariance of PA self-efficacy instruments in intervention studies is necessary to demonstrate that the same construct is measured over time with the same metric (Widaman et al., 2010). Unfortunately, psychometric assessment of PA self-efficacy instruments is rarely reported in the literature (Brown et al., 2009). This study aims to fill this gap by comparing the factorial validity, measurement invariance, and reliability of two PA self-efficacy instruments used in a large-scale study to test a PA intervention with urban, adolescent girls. The results may contribute to better understanding of PA self-efficacy and its role in fostering PA among girls.

The concept of PA self-efficacy has its origins within social cognitive theory (Bandura, 1986). Bandura (1997, p. 2) defines self-efficacy as ‘the belief in one’s capabilities to organize and execute courses of action required to produce given attainments.’ When this definition is applied to PA, self-efficacy is defined as a belief in one’s capability to participate in PA and to choose PA over existing barriers (Voskuil and Robbins, 2015). PA self-efficacy has been incorporated into several health behavior models used to develop theory-based interventions and explain PA in adolescents (Plotnikoff et al., 2013).

While several studies have included PA self-efficacy as a key construct in theory-based interventions with youth (Bauman et al., 2012), psychometric assessment of PA self-efficacy instruments has been insufficient. Brown et al. (2009) examined reporting of PA self-efficacy instrument validity and reliability in 15 studies. They noted that while the majority of studies included acceptable internal consistency with Cronbach’s alpha (*n* = 12), fewer than half reported reliability over time (*n* = 7), only two reported acceptable factor analyses, and none reported criterion validity. Furthermore, psychometric assessment of PA self-efficacy instruments utilized with specific populations, such as girls, has been limited (Dewar et al., 2013).

This study examines the potential for an intervention to alter the way in which participants understand and respond to a PA self-efficacy instrument. In the Trial of Activity for Adolescent Girls (TAAG), Lytle et al. (2009) reported that girls in the intervention group had lower self-efficacy scores at the end of the study compared to girls in the control group. These authors hypothesized that exposure to the intervention likely heightened girls’ awareness of their difficulties related to PA. Dunton et al. (2007) also reported declines in scores for PA self-efficacy among an intervention group of adolescent girls. Other researchers have noted similar findings for self-efficacy among youth, reporting lower PA self-efficacy after exposure to an intervention (Haerens et al., 2008; Bergh et al., 2012). Reporting mean differences in PA self-efficacy between an intervention and control group may be inaccurate if researchers assume measurement invariance without confirming it through invariance testing (Dishman et al., 2010).

Few studies have demonstrated support for the factorial validity and measurement invariance of PA self-efficacy instruments (Motl et al., 2000; Dishman et al., 2002, 2010; Roesch et al., 2013) with only the TAAG study demonstrating satisfactory cross-group and longitudinal invariance between intervention and control groups (Dishman et al., 2010). Roesch et al. (2013) established longitudinal invariance of a PA self-efficacy instrument measured in adolescents; however, the analysis did not separate the intervention and control groups. Additional investigation of longitudinal invariance of self-efficacy measures in intervention studies is warranted to better understand changes in the concept over time, influences by intervention effects, and effect on PA among adolescent girls.

Another concern regarding measurement of PA self-efficacy is that researchers often adapt established instruments without conducting psychometric analyses to confirm that their changes have not affected the measurement properties (Johnson et al., 2011; Bergh et al., 2012; Dewar et al., 2013). Deleting items, changing item wording, and altering response choices may have a significant impact on the reliability and validity of these instruments and can change the meaning of the underlying concept. For example, Sherwood et al. (2004) adapted a PA self-efficacy instrument for use with 8- to 10-year old girls by changing the main stem of item questions from *how sure are you* to *how hard do you think it would be.* The authors point out the modified items may have more accurately reflected perceived behavioral control than self-efficacy.

This study also aims to improve upon current reliability analysis for self-efficacy measures. Although frequently reported in psychometric studies (Brown et al., 2009), Cronbach’s alpha may underestimate the true reliability for scales with a limited number of items (Furr and Bacharach, 2014). Furthermore, alpha assumes that items are tau-equivalent, which is often not the case (Thurber and Bonynge, 2011). Assessing composite reliability via confirmatory factor analysis (CFA) may provide better support for internal consistency (Raykov, 2004) and be more accurate for multi-dimensional instruments than alpha (Barbaranelli et al., 2015). A specialized method for assessing composite reliabilty is also most appropriate for latent factors measured by ordinal Likert items with few response options (Yang and Green, 2011) of the sort typically used to assess self-efficacy among adolescents.

The purpose of this study was to compare the psychometric properties of two PA self-efficacy instruments used with urban 5th–8th grade girls in the “Girls on the Move” group randomized controlled trial (RCT; Robbins et al., 2013). The specific aims were to examine: (1) factorial validity; (2) multi-group and longitudinal invariance; and (3) composite reliability of the self-efficacy instruments in the group RCT’s control and intervention groups.

## Materials and Methods

### Design

The psychometric properties of two PA self-efficacy instruments were examined using secondary data from the first 2 years of the “Girls on the Move” group RCT. The group RCT was conducted to examine the effect of a 17-week multi-component intervention on increasing moderate-to-vigorous PA (MVPA) among racially diverse, underserved 5th–8th grade girls (Robbins et al., 2013). The group RCT included 24 urban schools in the Midwestern United States over three intervention years from 2012 to 2015. At baseline and again at the end of the 17-week intervention, girls completed an iPad-delivered survey that included the PA self-efficacy instruments. We used data collected during intervention years one and two of the group RCT to fulfill our aims.

### Sample and Setting

#### Sample

A total of 1,012 girls (*M* = 12.2; *SD* = 0.96) participated during the first two intervention years of the group RCT. Inclusion criteria for participants were: (1) 5th–7th grade girls (8th grade girls in schools with only 7th and 8th grades); (2) able to participate in a PA club 3 days a week after school; (3) anticipated availability to complete 9-month post-intervention follow-up measures; and (4) able to read, understand, and speak English. Girls were excluded if they had a health condition that prevented safe PA or were involved in after-school sports or a community program that included PA. Girls represented a diverse population with 526 (52.0%) blacks, 256 (25.3%) whites, and 133 (13.1%) mixed race with 108 (81.2%) of these girls selecting black as part of a mixed race. No significant differences between groups were found with the exception of race (χ^2^ = 6.385, *p* = 0.01) with more black girls in the control group. **Table 1** includes additional sample characteristics.

**Table 1:** Sample characteristics at baseline.

	Total sample (*N* = 1012)		Intervention (*N* = 510)		Control (*N* = 502)	
			
Characteristic	*n*	%	*n*	%	*n*	%
**Age (years)**						
10	90	8.9	48	9.4	42	8.4
11	373	36.9	180	35.3	193	38.4
12	343	33.9	184	36.1	159	31.7
13	163	16.1	78	15.3	85	16.9
14	43	4.2	20	3.9	23	4.6
15	4	0.4	2	0.4	2	0.4
**Grade**						
Fifth	69	6.8	34	6.7	35	7.0
Sixth	415	41.0	205	40.2	210	41.8
Seventh	412	40.7	214	42.0	198	39.4
Eighth	116	11.5	57	11.2	59	11.8
**Hispanic ethnicity**						
Yes	113	11.2	421	82.5	428	85.3
No	849	83.9	61	12.0	52	10.4
Not reported	50	0.5	28	5.5	22	4.4
**Race**						
Black^∗^	526	52.0	245	48.0	281	56.0
White	256	25.3	140	27.5	116	23.1
Mixed	133	13.1	68	13.3	65	12.9
Other	64	6.3	38	7.5	26	5.2
Not reported	33	3.3	19	3.7	14	2.8
**Free/reduced-price lunch^a^**						
Yes	804	79.4	392	76.9	412	82.1
No	136	13.4	75	14.7	61	12.2
Not reported	72	7.1	43	8.4	29	5.8
**Weight status**						
Underweight	10	1.0	4	0.8	6	1.2
Healthy weight	438	43.3	236	46.3	202	40.2
Overweight	226	22.3	115	22.5	111	22.1
Obese	338	33.3	155	30.4	183	36.5


*Percentages may not add to 100 due to rounding error. ^a^Free/reduced-price lunch program used as an indicator of socioeconomic status. ^∗^*p* < 0.05.*

#### Setting

Data for this study were collected in 16 schools. Eight schools, half of which served as controls, were involved in each of the two intervention years. School-level data indicated that the majority of girls in each school were black and of low socioeconomic status (SES), as determined by participation in the free or reduced-price lunch program. Schools were randomly assigned to receive either the intervention or control condition after baseline data collection. All school administrators, parents/guardians, and participants agreed to this randomization procedure.

### Measures

#### Demographics

Data on each girl’s age, grade, ethnicity, race, and participation in a free or reduced-price lunch program were collected via the consent form completed by girls’ parents/guardians.

#### Body Mass Index (BMI)

Body mass index (BMI) was included for use in describing the sample. Each girl’s measured weight and height were used to calculate BMI. Weight was measured to the nearest 0.1 kg using a foot-to-foot bioelectric impedance scale (Tanita Corporation, Tokyo, Japan). Height without shoes was measured to the nearest 0.1 cm using a Shorr Board^1^. BMI was calculated based on the formula of kg/meters^2^. BMI percentiles for age were calculated using the 2000 Centers for Disease Control (CDC) interpretation of BMI for children and teens (Centers for Disease Control and Prevention [CDC], 2015). Weight was classified as: (1) underweight (<5th percentile); (2) healthy weight (5th percentile to <85th percentile); (3) overweight (≥85th to <95th percentile); and (4) obese (≥95th percentile).

#### PA Self-Efficacy

Physical activity self-efficacy was measured using two instruments. The first was developed by Saunders et al. (1997) as a 17-item scale with three factors: support-seeking, barriers, and positive alternatives. Additional psychometric testing using CFA resulted in a unidimensional 8-item instrument that previously demonstrated multi-group and longitudinal invariance (Motl et al., 2000; Dishman et al., 2002, 2010). The revised instrument included items from each of the three factors identified by Saunders et al. (1997) with five response options ranging from (1) *disgaree a lot* to (5) *agree a lot* that replaced the dichotomous *yes/no* used initially.

This instrument was reduced to 6-items for use in the group RCT. Two social support items were excluded: (1) “*I can ask my parent or other adult to do physically active things with me*”; and (2) “*I can ask my best friend to be physically active with me during my free time on most days*.” The response choices were reduced from five to four to avoid a neutral response option: (0) *disagree a lot* to (3) *agree a lot*. Previous research suggests that eliminating a neutral response and offering four response choices may be optimal when surveying youth (Borgers et al., 2004). A sample item is “*I can be physically active in my free time on most days even when I am busy*.” We refer to the 6-item scale as PA Self-Efficacy (PASE).

The second instrument was developed as a 12-item Self-Efficacy for Exercise Behaviors Scale for use with adults (Sallis et al., 1988). Exploratory factor analysis (EFA) resulted in two factors, a 5-item resisting-relapse factor and a 7-item making-time-for-exercise factor. This instrument was further modified to a 10-item scale for use with adolescents and demonstrated adequate predictive validity (Wilson et al., 2002) and reliability (Wilson et al., 2008). Neither factorial validity nor measurement invariance testing of the instrument was found in the peer-reviewed literature. However, in an unpublished study, CFA did not support a unidimensional scale and showed inadequate fit to the data (Lawman et al., 2011). One item, “*How sure are you that you can stick to your exercise program when you are alone and no one is watching you*?” was found to be non-invariant between boys and girls and was deleted resulting in a 9-item scale.

In the group RCT, this instrument was revised to include 7-items with four response choices ranging from (0) *not at all sure* to (3) *very sure*. Two items were removed to reduce response burden and increase the relevance of items for adolescent girls: (1) “*How sure are you that you can stick to your exercising when you have guests staying in your home*?” and (2) “*How sure are you that you can stick to exercising even when you have limited amounts of time*? A sample item from the scale is “*How sure are you that you can stick to your exercise program even when your friends want to hang out*?” We refer to the 7-item scale as Self-Efficacy for Exercise Behaviors (SEEB).

### Procedures

#### Recruitment

The Michigan State University Institutional Review Board and school administrators provided approval to conduct the group RCT. Data collectors visited each school and community center to share information about the study with girls. Recruitment packets with study information, assent and consent forms, and an eligibility screening tool were provided to girls interested in participating. Girls were asked to share the packets with their parents or guardians and return completed packets to the researchers at their school within 2 days.

#### Data Collection

Eligible girls with signed consent and assent forms completed an iPad-delivered survey, including the PASE and SEEB, at baseline and after the 17-week intervention. Trained research assistants measured height and weight behind privacy screens. Details of the group RCT procedures have been reported elsewhere (Robbins et al., 2013).

### Data Analysis

Data analysis was performed using Stata 14 (StataCorp, 2015), Mplus 7.4 (Muthén and Muthén, 2015), and SAS 9.3 (SAS Institute Inc., 2011). Stata was used to calculate descriptive statistics of the sample, review characteristics of the PASE and SEEB items, assess missing data, and assess non-independence of the data. We used *t*-tests and chi-square tests to check for baseline differences between intervention and control groups.

The self-efficacy items for both instruments underwent a missing data analysis. Dummy variables for each of the self-efficacy items were created as dependent variables and a series of logistic regressions were performed to identify predictors of missingness. Although none of the demographic variables were found to predict missingness, we still included age, BMI, race, ethnicity, grade, school, pubertal status, and treatment group in the imputation model. All of the self-efficacy items were used as model predictors. The multiple imputation procedure with Stata 14 was used to impute the missing data using a single newly created data set. This decision was based on the fact that both instruments had <1% missing data at baseline and <10% missing data post-intervention, and this proportion was not likely to result in biased results (Dong and Peng, 2013). Each imputed value was a random draw from the conditional distribution of the variable being imputed given the observed values of the imputation predictors. Mean scores before and after imputation for all of the self-efficacy items were comparable with no significant differences found.

The potential for a clustering effect existed due to the group RCT multi-level structure with girls nested in schools. We computed item-specific intra-class correlation coefficients (ICCs) for the PASE and SEEB at both time points to assess non-independence of the data and ensure that single-level CFA was appropriate. Brown (2015) states that ICC values below 0.05 likely indicate that a multi-level CFA model may not be warranted. However, Musca et al. (2011) caution that even with ICC values as low as 0.01, Type I error rates can be greater than 5%. Of the 26 ICC values, the majority were close to zero. Only three items had ICCs > 0.01. The highest ICC value was 0.013 for one of the SEEB items at baseline. Given the low ICCs for both instruments at both time points and small number of schools in the sample, we decided to conduct invariance testing using single-level CFA. We doubt that a multilevel CFA is computationally feasible with so little school-level variance in the indicators and that trustworthy parameter estimates could be obtained from a school-level covariance matrix representing data from only 16 schools.

We ran CFA models using Mplus to determine factorial validity and measurement invariance of the PASE and SEEB instruments. Parameters were estimated using weighted least squares with mean and variance adjustment (WLSMV) with delta parameterization in which data are fitted to a polychoric correlation matrix. This estimation method is recommended for ordinal indicators with fewer than five response choices and is also robust to skewness and kurtosis of items (Flora and Curran, 2004; Flora et al., 2012; Brown, 2015). We scaled each latent factor by fixing the factor loading for the first indicator to 1. We chose referent indicators by selecting items with the greatest variability and satisfactory standardized parameter estimates (Johnson et al., 2009).

Our invariance testing began with an assessment of separate single group models for the intervention and control groups at each time point for both instruments. Analysis then proceeded to cross-group measurement invariance between the intervention and control groups as well as longitudinal invariance for each group from baseline to post-intervention. The last step involved setting *simultaneous* cross-group and longitudinal constraints on parameters. Invariance analysis was conducted following guidelines from Muthén and Muthén (2015) for ordinal data using WLSMV estimation and included: (1) configural invariance – non-referent factor loadings and all thresholds free for both groups/time points, scale factors fixed at one for both groups/time points, factor means fixed at zero for both groups/time points, and factor variances free for both groups/time points; (2) metric invariance – non-referent factor loadings set equal for both groups/time points, scale factors fixed at one for the control group/at baseline and free for the intervention group/at post-intervention, factor means fixed at zero for the control group/at baseline and free for the intervention group/at post-intervention, first threshold of each indicator set equal for both groups/time points as well as the second threshold of the referent indicator to identify the latent factor mean, and factor variances free for both groups/time points; and (3) scalar invariance – non-referent factor loadings and all thresholds set equal for both groups/time points, scale factors fixed at one for the control group/at baseline and free for the intervention group/at post-intervention, factor means fixed at zero for the control group/at baseline and free for the intervention group/at post-intervention, and factor variances free for both groups/time points.

Because invariant factor loadings are vital for construct validity (Brown, 2015), partial metric invariance was not undertaken with the separate cross-group and longitudinal invariance models. Instead, if the metric model resulted in non-invariant factor loadings, we re-specified the model. However, we pursued partial metric invariance for the final *simultaneous* invariance models to determine which factor loadings were non-invariant. We explored partial scalar (threshold) invariance by examining threshold differences between groups or time points as well as suggested modification indices (MIs). We used guidelines from Coertjens et al. (2012) to explore partial threshold invariance as well as Dimitrov (2010) who suggested that partial invariance might be satisfactory if <20% of parameters are non-invariant.

We used the model chi-square test to evaluate initial fit in single group models as well as measurement invariance models. Because this test can be sensitive to large sample sizes, we also used alternative fit indices for model evaluation, including the comparative fit index (CFI) and the root mean square of approximation (RMSEA). Per recommendations from Kline (2016) and Brown (2015), we used the following guidelines to assess model fit: CFI ≥ 0.95; RMSEA ≤ 0.05 for close fit, ≤0.08 for approximate fit, >0.08 to <0.1.0 for marginal fit, and ≥1.0 for poor fit, and evaluation of lower- and upper-bound RMSEA 90% confidence intervals. Many researchers using structural equation models or CFA ignore significant chi-square tests as evidence against a model, citing reasons such as the sensitivity of the test to large samples (Ropovik, 2015). Brown (2015), Ropovik (2015), and Kline (2016) argue against blithely accepting models solely on the basis of other global fit indices when the chi-square is significant, advising careful assessment of other evidence regarding model tenability first. Therefore, when the chi-square test was significant, we used recommendations from these authors to identify localized areas of strain by closely evaluating the correlation residuals and MIs. For this study, when the majority of correlated residuals were <0.10 (Kline, 2016), MIs were small, and alternative fit indices indicated acceptable fit, measurement invariance continued based on the fact that these models were considered plausible (Byrne et al., 1989; Raykov et al., 2012).

After evaluating single group models, we evaluated measurement invariance via chi-square difference testing between baseline and nested models. A corrected chi-square difference test was used because the differences are not distributed as chi-square using WLSMV (Brown, 2015). We evaluated RMSEA and CFI fit indices at each step of invariance testing, along with change in CFI. Cheung and Rensvold (2002) recommend using a change in CFI between models ≥-0.01 as potentially indicating non-invariance. However, Meade et al. (2008) suggested a CFI change of >-0.002 as an indication of non-invariance. Therefore, we interpreted changes in CFI with caution because these guidelines were based on simulation studies using maximum likelihood estimation with normally distributed data. These cutoffs have not been evaluated with WLSMV estimation and ordinal data.

Cronbach’s alpha assumes tau-equivalence (equal factor loadings across items within a scale), often underestimates reliability for scales with few items, and is inappropriate for ordinal Likert scale data (Yang and Green, 2011; Furr and Bacharach, 2014). Composite reliability of the instruments was therefore estimated via Green and Yang’s (2009) non-linear structural equation modeling (SEM) reliability coefficient, which is based on a parallel-forms definition of reliability. This coefficient is designed for latent factors measured by ordinal indicators (Green and Yang, 2009; Yang and Green, 2015). Kelley and Pornprasertmanit (2016) called this coefficient “categorical omega” to differentiate it from the more commonly used omega coefficient, which uses linear SEM parameter estimates to compute reliability and assumes that the indicators are continuous. Simulations show that Green and Yang’s coefficient performs as well as or better than either omega or Cronbach’s alpha in a variety of conditions (Green and Yang, 2009; Yang and Green, 2011; Yang and Green, 2015). Computations occurred in three steps: (1) estimation of thresholds and polychoric correlations; (2) fitting the CFA model to the polychoric correlation matrix using WLSMV; and (3) inputting factor loadings and thresholds into the equation using a SAS program to calculate the reliability coefficient. Steps one and two were conducted in Mplus. Results were transferred to SAS to carry out step 3.

## Results

### Descriptive Statistics of Items

We ran item-level analyses for each indicator of the PASE and SEEB instruments at baseline and post-intervention for the full sample and separately in intervention and control groups. **Table 2** includes item descriptions for the PASE and SEEB. Descriptive statistics and polychoric correlations of items can be found in the Supplementary Material.

**Table 2:** Survey instrument item descriptions.

Item	PASE item description
1	I can be active in my free time on most days.
2	I can be active in my free time on most days instead of watching TV or playing video games.
3	I can be active or play active games or sports in my free time on most days when it is hot or cold out.
4	I can be active in my free time on most days when I have to stay home.
5	I have the skills I need to be active in my free time on most days.
6	I can be active in my free time on most days even when I am busy.

**Item**	**SEEB item description**

1	How sure are you that you can stick to your exercise program when your family is demanding more time from you?
2	How sure are you that you can stick to your exercise program when you have household chores?
3	How sure are you that you can stick to your exercising when you’re feeling lazy?
4	How sure are you that you can stick to participating in activities that include exercise?
5	How sure are you that you can stick to your exercise program even when your friends want to hang out?
6	How sure are you that you can stick to making exercise a top priority?
7	How sure are you that you can stick to your exercise program when you have a lot of demands at school?


*PASE, Physical Activity Self-Efficacy Scale; SEEB, Self-Efficacy for Exercise Behaviors Scale.*

Overall, girls primarily selected *agree a little* or *agree a lot* responses for the PASE items, yielding skewed distributions and ceiling effects. The exception was the item, “*I can be active in my free time on most days even when I am busy*” in which girls’ responses had greater variability. Mean inter-item polychoric correlations for the full sample were 0.44 (minimum–maximum: 0.38–0.58) and 0.52 (minimum–maximum: 0.46–0.63) at baseline and post-intervention, respectively. Mean scores for the PASE for the full sample were 2.20 (*SD* = 0.59) at baseline and 2.17 (*SD* = 0.59) post-intervention.

Compared to the PASE, the SEEB items had lower mean scores and were less skewed with girls being more likely to endorse the 0 and 1 response options (i.e., *not at all sure* or *not very sure*). However, 1-item, “*How sure are you that you can stick to participating in activities that include exercise?*” had over 50% of girls endorsing the highest response option of *very sure* at baseline. This item had the highest mean score of the SEEB items with limited variance and marked skewness and kurtosis. Mean inter-item polychoric correlations for the full sample were 0.39 (minimum–maximum: 0.30–0.51) and 0.41 (minimum–maximum: 0.29–0.57) at baseline and post-intervention, respectively. For the full sample, the SEEB mean score at baseline was 1.90 (*SD* = 0.59) and 1.82 (*SD* = 0.60) at post-intervention.

### Measurement Invariance

#### Cross-Group Invariance

**FIGURE 1:**
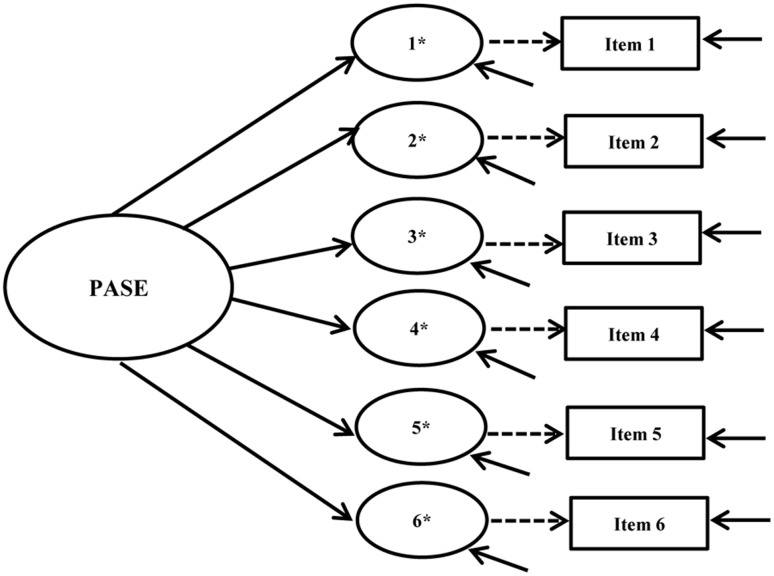
Physical Activity Self-Efficacy Scale (PASE) hypothesized path diagram. Figure includes the latent factor, underlying latent response variables indicated by an ^∗^, and observed indicators. Small solid arrows denote disturbance and error variances for the latent response variables and observed indicators, respectively.

**FIGURE 2:**
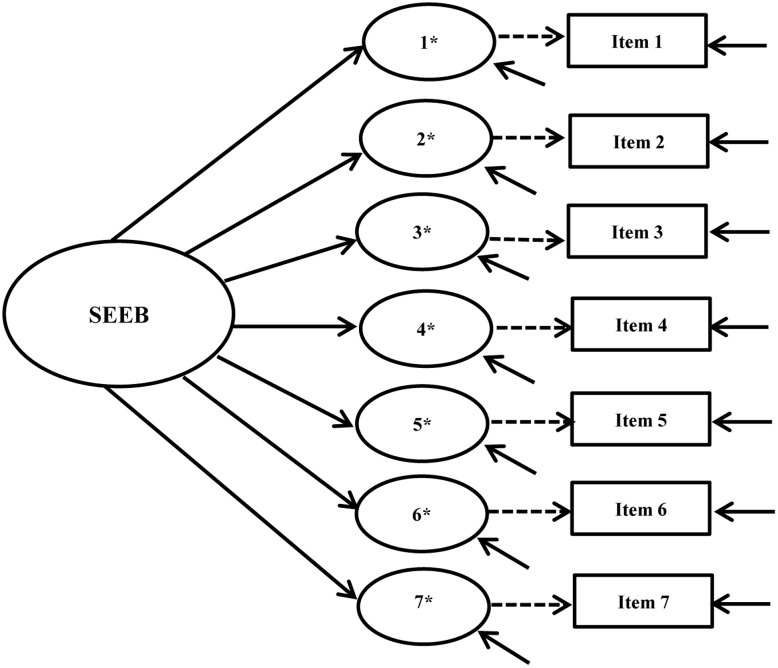
Self-Efficacy for Exercise Behaviors Scale (SEEB) hypothesized path diagram. Figure includes the latent factor, underlying latent response variables indicated by an ^∗^, and observed indicators. Small solid arrows denote disturbance and error variances for the latent response variables and observed indicators, respectively.

Our examination of cross-group measurement invariance began with single group, cross-sectional CFA models for the intervention and control groups at baseline and post-intervention using pre-specified fit criteria. Configural, metric, and scalar invariance tests were conducted following this analysis. **Figures 1**, **2** show the hypothesized path diagrams for the PASE and SEEB. Parameter estimates, including factor loadings, thresholds, and *r*-square values for each instrument, are summarized in the first author’s dissertation (Voskuil, 2016). Model results are presented in **Table 3**.

**Table 3:** Cross-group measurement invariance results.

Model	χ^2^ *(df)*	*p*	χ^2^_diff_	Δ*df*	*p*	RMSEA [90% CI]	CFI	ΔCFI
**PASE baseline**							
INT	8.091 (9)	0.525	–	–	–	0.000 [0.000, 0.046]	1.000	–
CON	32.503 (9)	<0.001	–	–	–	0.072 [0.046, 0.100]	0.984	–
MI								
M1	39.438 (18)	0.003	–	–	–	0.049 [0.028, 0.069]	0.993	–
M2	43.780 (23)	0.006	7.849	5	0.165	0.042 [0.022, 0.061]	0.993	0.000
M3	76.490 (34)	<0.001	35.009	11	<0.001	0.050 [0.035, 0.065]	0.986	-0.007
M4	67.642 (33)	<0.001	25.850	10	0.004	0.046 [0.030, 0.061]	0.988	-0.005
M5	61.474 (32)	0.001	18.472	9	0.030	0.043 [0.026, 0.059]	0.990	-0.003
M6	55.945 (31)	0.004	12.840	8	0.118	0.040 [0.022, 0.056]	0.992	-0.001
**PASE post-intervention**							
INT	10.660 (9)	0.300	–	–	–	0.019 [0.000, 0.055]	0.999	–
CON	39.893 (9)	<0.001	–	–	–	0.083 [0.058, 0.110]	0.978	–
MI								
M1	53.591 (18)	<0.001	–	–	–	0.063 [0.044, 0.082]	0.991	–
M2	54.132 (23)	<0.001	6.933	5	0.226	0.052 [0.034, 0.070]	0.992	0.001
M3	82.580 (34)	<0.001	30.008	11	0.002	0.053 [0.039, 0.068]	0.987	-0.005
M4	72.345 (33)	<0.001	19.969	10	0.030	0.049 [0.033, 0.064]	0.990	-0.002
M5	65.702 (32)	<0.001	13.456	9	0.143	0.046 [0.030, 0.061]	0.991	-0.001
**SEEB^a^ baseline**							
INT	10.525 (5)	0.062	–	–	–	0.047 [0.000, 0.086]	0.995	–
CON	6.855 (5)	0.232	–	–	–	0.027 [0.000, 0.072]	0.998	–
MI								
M1	17.234 (10)	0.069	–	–	–	0.038 [0.000, 0.067]	0.996	–
M2	22.225 (14)	0.074	6.046	4	0.196	0.034 [0.000, 0.060]	0.996	0.000
M3	34.651 (23)	0.056	12.791	9	0.172	0.032 [0.000, 0.052]	0.994	-0.002
**SEEB^a^ post-intervention**							
INT	22.103 (5)	0.001	–	–	–	0.082 [0.049, 0.118]	0.990	–
CON	24.990 (5)	<0.001	–	–	–	0.089 [0.056, 0.125]	0.979	–
MI								
M1	47.181 (10)	<0.001	–	–	–	0.086 [0.062, 0.111]	0.986	–
M2	36.543 (14)	0.001	2.184	4	0.702	0.056 [0.034, 0.079]	0.992	0.006
M3	54.144 (23)	<0.001	18.974	9	0.025	0.052 [0.034, 0.070]	0.988	-0.004
M4	40.958 (22)	0.008	5.650	8	0.686	0.041 [0.021, 0.061]	0.993	0.005


*χ^*2*^_diff_, adjusted χ^*2*^ difference test used to compare models; *df*, degrees of freedom; PASE, Physical Activity Self-Efficacy Scale; SEEB, Self-Efficacy for Exercise Behaviors Scale; RMSEA, root mean square error of approximation; 90% CI, 90% confidence interval for RMSEA; CFI, comparative fit index; *Δ*CFI, change in comparative fit index; INT, intervention group; CON, control group; MI, measurement invariance models; M1, configural model; M2, metric model; M3, scalar model; M4, M5, M6, partial scalar models. ^a^SEEB model results from modified 5-item scale without items 2 and 3.*

##### Physical Activity Self-Efficacy Scale (PASE)

CFA models for the intervention group demonstrated an excellent fit to the data at baseline (χ^2^ = 8.091, *df* = 9, *p* = 0.525, RMSEA = 0.000, CFI = 1.000) and post-intervention (χ^2^ = 10.660, *df* = 9, *p* = 0.300, RMSEA = 0.019, CFI = 0.999). Based on RMSEA and CFI values, model fit for the control group was acceptable at baseline (χ^2^ = 32.503, *df* = 9, *p* < 0.001, RMSEA = 0.072, CFI = 0.984) and marginal at post-intervention (χ^2^ = 39.893, *df* = 9, *p* < 0.001, RMSEA = 0.083, CFI = 0.978).

Given the significant chi-square and higher RMSEA values in the control group, we looked for areas of strain at both time points. All residual correlations at baseline were <0.10, but at post-intervention one residual correlation >0.10 was noted between items 3 and 4 (-0.106) in the control group. Suggested MIs at both time points were low in value, not substantively justifiable, and not indicated in the intervention group. Therefore, cross-group invariance testing continued because the models appeared plausible. Others have suggested that baseline models may not need to entirely meet pre-determined fit criteria if the model appears reasonable (Byrne et al., 1989; Raykov et al., 2012; Bowen and Masa, 2015).

Fit indices, with the exception of the model chi-square test, supported configural invariance between groups at both time points (baseline: χ^2^ = 39.438, *df* = 18, *p* = 0.003, RMSEA = 0.049, CFI = 0.993; post-intervention: χ^2^ = 53.591, *df* = 18, *p* < 0.001, RMSEA = 0.063, CFI = 0.991). All residual correlations were <0.10 for both groups at both time points with the exception of the previously mentioned correlated residual. Similar to the configural model, all residual correlations for the metric model were <0.10 with the exception of one residual correlation at post-intervention between items 3 and 4 (-0.126) in the control group. Metric invariance between groups was supported at baseline (Δχ^2^ = 7.849, Δ*df* = 5, *p* = 0.165, RMSEA = 0.042, CFI = 0.993, ΔCFI = 0.000) and post-intervention (Δχ^2^ = 6.933, Δ*df* = 5, *p* = 0.226, RMSEA = 0.052, CFI = 0.992, ΔCFI = 0.001).

Scalar invariance between groups was not supported at either time point indicating non-invariant thresholds across groups (baseline: Δχ^2^ = 35.009, Δ*df* = 11, *p* < 0.001, RMSEA = 0.050, CFI = 0.986, ΔCFI = -0.007; post-intervention: Δχ^2^ = 30.008, Δ*df* = 11, *p* = 0.002, RMSEA = 0.053, CFI = 0.987, ΔCFI = -0.005). We examined MIs and expected parameter change (EPC) values to see which threshold might be freed in an attempt to achieve partial scalar invariance. We revised scalar invariance models by freeing these thresholds one at a time starting with the threshold with the largest modification index, and freeing additional thresholds if scalar invariance was not achieved (Dimitrov, 2010; Coertjens et al., 2012).

At baseline, partial scalar invariance testing began by freeing the third threshold (going from *agree a little* to *agree a lot*) for item 3. However, this approach did not improve model fit enough to achieve invariance (Δχ^2^ = 25.850, Δ*df* = 10, *p* = 0.004, RMSEA = 0.046, CFI = 0.988, ΔCFI = -0.005). The process was repeated by freeing the first threshold of item 1 (going from *disagree a lot* to *disagree a little*), but invariance was still not met (Δχ^2^ = 18.472, Δ*df* = 9, *p* = 0.030, RMSEA = 0.043, CFI = 0.990, ΔCFI = -0.003). After freeing the third threshold of item 2, partial scalar invariance was met (Δχ^2^ = 12.840, Δ*df* = 8, *p* = 0.118, RMSEA = 0.040, CFI = 0.992, ΔCFI = -0.001). Thus 3 of 18 thresholds were non-invariant (16.7%) with 3 of 6-items still having fully invariant thresholds. All residual correlations for this partial threshold invariance model were <0.10 and alternative fit indices demonstrated good fit to the data.

Testing for post-intervention scalar invariance proceeded in the same manner. We began by freeing the third threshold for item 2. This action still resulted in scalar non-invariance (Δχ^2^ = 19.969, Δ*df* = 10, *p* = 0.030, RMSEA = 0.049, CFI = 0.990, ΔCFI = -0.002). MIs and EPC values indicated that the second threshold (going from *disagree a little* to *agree a little*) for item 6 should also be freed. Doing so improved model fit and supported partial scalar invariance (Δχ^2^ = 13.456, Δ*df* = 9, *p* = 0.143, RMSEA = 0.046, CFI = 0.991, ΔCFI = -0.001). Despite the significant chi-square value for this model, all residual correlations were <0.10 with the exception of the previously mentioned residual correlation between items 3 and 4 of -0.126. Alternative fit indices demonstrated good fit to the data.

##### Self-Efficacy for Exercise Behaviors Scale (SEEB)

Initial models for the intervention group (χ^2^ = 74.255, *df* = 14, *p* < 0.001, RMSEA = 0.092, CFI = 0.967) and control group (χ^2^ = 58.828, *df* = 14, *p* < 0.001, RMSEA = 0.080, CFI = 0.966) at baseline showed a marginal fit to the data. Model fit was poor in the intervention group (χ^2^ = 124.709, *df* = 14, *p* < 0.001, RMSEA = 0.125, CFI = 0.951) and control group (χ^2^ = 84.340, *df* = 14, *p* < 0.001, RMSEA = 0.100, CFI = 0.952) at post-intervention.

Consistent areas of strain across groups and time points indicated the need to add a residual covariance between items 1 and 2. Adding a residual covariance for these two items was theoretically justifiable given the connection to family responsibilities for each of these items. Another area of strain in the control group at baseline and post-intervention was a residual correlation between items 2 and 6. However, this residual correlation was not present in the intervention group at either time point so we chose not to add this to the model.

The adjusted models improved fit: (1) intervention group at baseline: χ^2^ = 26.042, *df* = 13, *p* = 0.017, RMSEA = 0.044, CFI = 0.993; (2) control group at baseline: χ^2^ = 41.407, *df* = 13, *p* < 0.001, RMSEA = 0.066, CFI = 0.978; (3) intervention group post-intervention: χ^2^ = 66.676, *df* = 13, *p* < 0.001, RMSEA = 0.090, CFI = 0.976 and (4) control group post-intervention: χ^2^ = 46.905, *df* = 13, *p* < 0.001, RMSEA = 0.072, CFI = 0.977). Areas of strain were as follows: (1) control group at baseline: a residual correlation >0.10 between items 2 and 5 (-0.113); and (2) intervention group post-intervention: a residual correlation >0.10 between items 4 and 6 (-0.104).

Compared to the PASE in which intervention group models fit better than control group models, the SEEB models fit the data better in both groups at baseline vs. post-intervention. Although the chi-square values were all statistically significant, none of the models indicated poor fit according to the pre-specified RMSEA and CFI criteria. Therefore, cross-group measurement invariance was undertaken given that these models appeared plausible. The results confirmed invariant factor loadings (Δχ^2^ = 5.495, Δ*df* = 6, *p* = 0.482, RMSEA = 0.064, CFI = 0.983, ΔCFI = 0.006) and thresholds (Δχ^2^ = 21.967, Δ*df* = 13, *p* = 0.056, RMSEA = 0.056, CFI = 0.981, ΔCFI = -0.002) post-intervention across groups, but factor loadings were not invariant at baseline (Δχ^2^ = 14.636, Δ*df* = 6, *p* = 0.023, RMSEA = 0.054, CFI = 0.985, ΔCFI = -0.002).

Each item was tested one at a time to determine which factor loadings were non-invariant at baseline. When the factor loading for item 3, “*How sure are you that you can stick to exercising when you’re feeling lazy*,” was unconstrained, metric invariance was achieved. For this study, partial metric invariance was not considered acceptable because it was assumed to be a fundamental requirement for determining that the same construct is being measured across groups and over time (Coertjens et al., 2012).

Several re-specifications of the initial hypothesized model were attempted. First, single group models were re-specified by dropping the above non-invariant item. It is worth noting that item 3 was not part of the original psychometric development study for this scale (Sallis et al., 1988). This item also had consistently lower factor loadings than the other items. Therefore, single group models excluding this item were analyzed. Again, baseline model fit was acceptable for both groups (intervention: χ^2^ = 14.906, *df* = 8, *p* = 0.060, RMSEA = 0.041, CFI = 0.996; control: χ^2^ = 20.779, *df* = 8, *p* = 0.008, RMSEA = 0.056, CFI = 0.988) but marginal at post-intervention for both groups (intervention: χ^2^ = 43.522, *df* = 8, *p* < 0.001, RMSEA = 0.093, CFI = 0.983; control: χ^2^ = 46.723, *df* = 8, *p* < 0.001, RMSEA = 0.098, CFI = 0.968).

We then tested a 5-item model after dropping items 2 and 3 as both demonstrated residual correlations >0.10 with other items. Both of these items also consistently had the lowest factor loadings and *R*^2^-values across models, particularly at post-intervention for both groups. Dropping item 2 also eliminated the need for the residual covariance between items 1 and 2. All further analyses of models for this instrument were conducted using this 5-item model.

Single group results for this 5-item model improved fit in the control (χ^2^ = 6.855, *df* = 5, *p* = 0.232, RMSEA = 0.027, CFI = 0.998) and intervention (χ^2^ = 10.525, *df* = 5, *p* = 0.062, RMSEA = 0.047, CFI = 0.995) groups at baseline. Overall fit post-intervention was still inferior to baseline fit. However, dropping the items yielded lower overall chi-square values, lower RMSEA values approaching acceptable model fit, higher CFI values, and no residual correlations ≥0.10 in the control group (χ^2^ = 24.990, *df* = 5, *p* < 0.001, RMSEA = 0.089, CFI = 0.979) or intervention group (χ^2^ = 22.103, *df* = 5, *p* < 0.001, RMSEA = 0.082, CFI = 0.990).

Configural invariance at baseline demonstrated good fit to the data (χ^2^ = 17.234, *df* = 10, *p* = 0.069, RMSEA = 0.038, CFI = 0.996). Both metric (Δχ^2^ = 6.046, Δ*df* = 4, *p* = 0.196, RMSEA = 0.034, CFI = 0.996, ΔCFI = 0.000) and scalar (Δχ^2^ = 12.791, Δ*df* = 9, *p* = 0.172, RMSEA = 0.032, CFI = 0.994, ΔCFI = -0.002) invariance were established across groups. Thus, the factor loadings and thresholds for the 5-item model were both invariant at baseline.

Fit indices for the post-intervention configural model provided some support for equal form across groups but it did not fit the data as well as the baseline model: χ^2^ = 47.181, *df* = 10, *p* < 0.001, RMSEA = 0.086, CFI = 0.986. All residual correlations were <0.10. A modification index of 19.795 suggested a residual covariance between items 1 and 4 but was neither theoretically justified nor indicated at baseline so we did not make this change. Because the RMSEA approached acceptable fit and the CFI was acceptable, metric and scalar invariance were also assessed at post-intervention. The metric model indicated invariant factor loadings when compared to the configural model (Δχ^2^ = 2.184, Δ*df* = 4, *p* = 0.702, RMSEA = 0.056, CFI = 0.992, ΔCFI = 0.006). However, scalar invariance of thresholds was not met (Δχ^2^ = 18.974, Δ*df* = 9, *p* = 0.025, RMSEA = 0.052, CFI = 0.988, ΔCFI = -0.004). All residual correlations were <0.10 except that between items 1 and 4 in the control group (0.115).

We investigated SEEB partial scalar invariance using the same process described for the PASE. MIs and EPC values pointed to the thresholds for item 4, “*How sure are you that you can stick to participating in activities that include exercise*,” as potentially non-invariant. The third threshold (going from *somewhat sure* to *very sure*) had the largest difference between groups. Partial scalar invariance was met by freeing it: Δχ^2^ = 5.650, Δ*df* = 8, *p* = 0.686, RMSEA = 0.041, CFI = 0.993, ΔCFI = 0.001. All residual correlations were <0.10 except between items 1 and 4 in the control group (0.116). One of 15 thresholds was non-invariant (6.7%) with 4 of 5-items still having fully invariant thresholds.

#### Longitudinal Invariance

**FIGURE 3:**
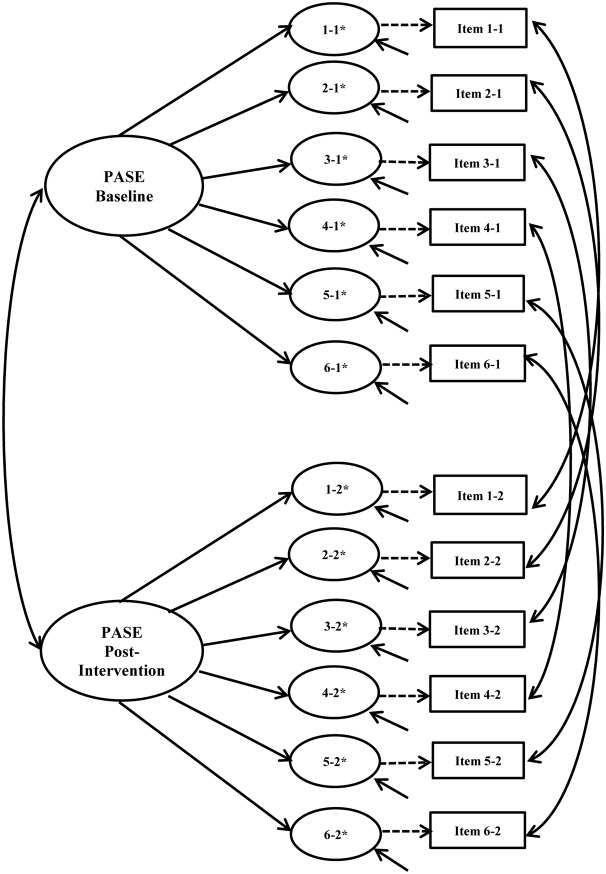
Physical Activity Self-Efficacy Scale (PASE) hypothesized longitudinal measurement model. Figure includes the latent factor, underlying latent response variables indicated by an ^∗^, and observed indicators. Small solid arrows denote disturbance and error variances for the latent response variables and observed indicators, respectively.

**FIGURE 4:**
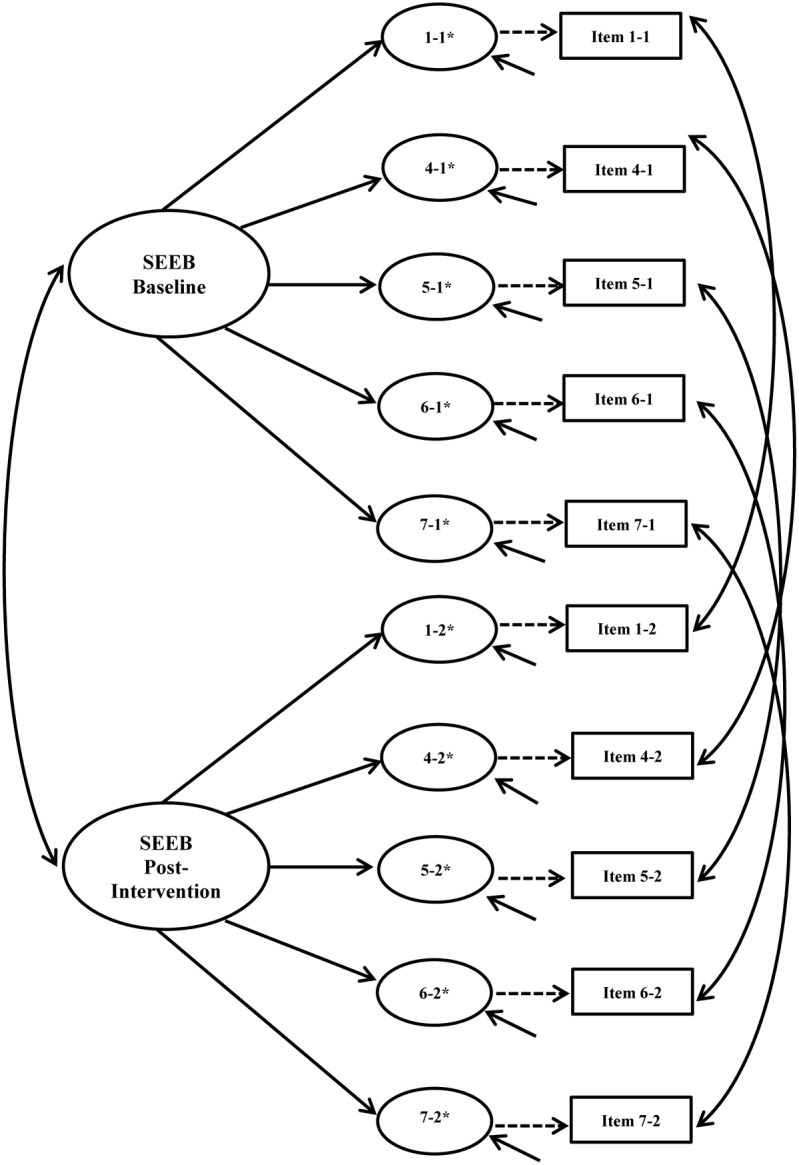
Self-Efficacy for Exercise Behaviors Scale (SEEB) hypothesized longitudinal measurement model. Figure includes the latent factor, underlying latent response variables indicated by an ^∗^, and observed indicators. Small solid arrows denote disturbance and error variances for the latent response variables and observed indicators, respectively.

Longitudinal invariance was assessed for each instrument. Error covariances for each item across time points were built into the model to account for expected method effects for repeated measures (Brown, 2015). **Figures 3**, **4** show the hypothesized longitudinal measurement models for the PASE and SEEB. **Figure 4** is based on the 5-item revised measurement model for the SEEB. Fit indices are shown in **Table 4**. Parameter estimates for the CFA models are provided in the first author’s dissertation (Voskuil, 2016).

**Table 4:** Longitudinal invariance results.

Model	χ^2^ (*df)*	*p*	χ^2^_diff_	Δ*df*	*p*	RMSEA [90% CI]	CFI	ΔCFI

PASE							
**Intervention group**							
M1	52.418 (47)	0.272	–	–	–	0.015 [0.000, 0.034]	0.999	–
M2	64.074 (52)	0.122	10.398	5	0.065	0.021 [0.000, 0.037]	0.997	-0.002
M3	89.557 (63)	0.016	28.693	11	0.003	0.029 [0.013, 0.042]	0.993	-0.004
M4	77.903 (62)	0.084	14.845	10	0.138	0.022 [0.000, 0.037]	0.996	-0.001
**Control group**							
M1	98.550 (47)	<0.001	–	–	–	0.047 [0.034, 0.060]	0.984	–
M2	103.012 (52)	<0.001	7.128	5	0.211	0.044 [0.032, 0.057]	0.984	0.000
M3	119.830 (63)	<0.001	18.306	11	0.075	0.042 [0.031, 0.054]	0.982	-0.002

**SEEB^a^**							

**Intervention group**							
M1	62.631 (29)	<0.001	–	–	–	0.048 [0.031, 0.064]	0.987	–
M2	68.819 (33)	<0.001	7.194	4	0.126	0.046 [0.031, 0.061]	0.987	0.000
M3	98.944 (42)	<0.001	34.267	9	<0.001	0.052 [0.038, 0.065]	0.979	-0.008
M4	71.630 (41)	0.002	0.929	8	0.999	0.038 [0.023, 0.053]	0.988	0.001
**Control group**							
M1	52.819 (29)	0.004	–	–	–	0.040 [0.022, 0.058]	0.987	–
M2	49.598 (33)	0.032	0.655	4	0.957	0.032 [0.010, 0.049]	0.991	0.004
M3	61.749 (42)	0.025	12.653	9	0.179	0.031 [0.011, 0.046]	0.990	-0.001


*χ^*2*^_diff_, adjusted χ^*2*^ difference test; *df*, degrees of freedom; PASE, Physical Activity Self-Efficacy Scale; SEEB, Self-Efficacy for Exercise Behaviors Scale; RMSEA, root mean square error of approximation; 90% CI, 90% confidence interval for RMSEA; CFI, comparative fit index; *Δ*CFI, change in comparative fit index; M1, configural model; M2, metric model; M3, scalar model; M4, partial scalar model. ^a^SEEB model results from modified 5-item scale without items 2 and 3.*

##### Physical Activity Self-Efficacy Scale (PASE)

The PASE configural model for the intervention group over time demonstrated excellent fit to the data (χ^2^ = 52.418, *df* = 47, *p* = 0.272, RMSEA = 0.015, CFI = 0.999). The metric model also fit the data well and indicated invariant factor loadings over time (χ^2^ = 64.074, *df* = 52, *p* = 0.122, Δχ^2^ = 10.398, Δ*df* = 5, *p* = 0.065, RMSEA = 0.021, CFI = 0.997, ΔCFI = -0.002). Scalar invariance was not supported (χ^2^ = 89.557, *df* = 63, *p* = 0.016, Δχ^2^ = 28.693, Δ*df* = 11, *p* = 0.003, RMSEA = 0.029, CFI = 0.993, ΔCFI = -0.004). We checked for partial scalar invariance with the same process used in the cross-group models. Freeing the third threshold for item 5 at both time points resulted in partial scalar invariance (χ^2^ = 77.903, *df* = 62, *p* = 0.084, Δχ^2^ = 14.845, Δ*df* = 10, *p* = 0.138, RMSEA = 0.022, CFI = 0.996, ΔCFI = -0.001).

The configural model for the control group resulted in a significant chi-square value (χ^2^ = 98.550, *df* = 47, *p* < 0.001). Two correlated residuals >0.10 resulted: item 6 at baseline with item 3 at post-intervention (0.118) and items 3 and 4 at post-intervention (-0.111). We proceeded with metric invariance testing because: (1) areas of strain were not present in the intervention group; (2) freeing the parameters with large MIs was not theoretically justified; and (3) alternative fit indices supported adequate fit. Results for the control group were consistent with longitudinally invariant factor loadings (Δχ^2^ = 7.128, Δ*df* = 5, *p* = 0.211, RMSEA = 0.044, CFI = 0.984, ΔCFI = 0.000). Scalar invariance was also supported in the control group (Δχ^2^ = 18.306, Δ*df* = 11, *p* = 0.075, RMSEA = 0.042, CFI = 0.982, ΔCFI = -0.002), indicating longitudinally invariant thresholds. All residual correlations for both the metric and scalar models were <0.10 except the two described above.

##### Self-Efficacy for Exercise Behaviors Scale (SEEB)

Similar to the PASE longitudinal results, the configural models for both groups resulted in significant model chi-square values. Areas of strain were assessed in both groups at both time points. For the control group, one correlated residual ≥0.10 was noted: item 4 at baseline with item 5 at post-intervention (-0.102). The intervention group also had one residual correlation ≥0.10 as follows: item 4 at baseline with item 6 at post-intervention (0.106). Alternative fit indices demonstrated adequate fit to the data with RMSEA values <0.08 and CFI values >0.95, so we proceeded to test metric models without freeing any additional parameters.

Longitudinal metric invariance was supported for the intervention group (Δχ^2^ = 7.194, Δ*df* = 4, *p* = 0.126, RMSEA = 0.046, CFI = 0.987, ΔCFI = 0.000), but scalar invariance was not (Δχ^2^ = 34.267, Δ*df* = 9, *p* < 0.001, RMSEA = 0.052, CFI = 0.979, ΔCFI = -0.008). MIs, EPC values, and the differences in threshold values again indicated that the third threshold of item 4 may vary over time. We achieved partial longitudinal scalar invariance by freeing this threshold (Δχ^2^ = 0.929, Δ*df* = 8, *p* = 0.999, RMSEA = 0.038, CFI = 0.988, ΔCFI = 0.001). All residual correlations for the metric, scalar, and partial scalar models were <0.10 except that described above between item 4 at baseline and item 6 at post-intervention. Metric and scalar invariance were also supported for the control group (metric: Δχ^2^ = 0.655, Δ*df* = 4, *p* = 0.957, RMSEA = 0.032, CFI = 0.991, ΔCFI = 0.004; scalar: Δχ^2^ = 12.653, Δ*df* = 9, *p* = 0.179, RMSEA = 0.031, CFI = 0.990, ΔCFI = -0.001). All residual correlations were <0.10 for the metric and scalar models with the exception of item 4 at baseline with item 5 at post-intervention (-0.100) for the metric model.

#### Simultaneous Cross-Group and Longitudinal Invariance

For each instrument, we then tested for simultaneous cross-group, longitudinal invariance by constraining parameters in multiple ways. For example, to test metric invariance, factor loadings at baseline and post-intervention were constrained to be equal in the control group model, as were intervention group loadings. Results for both instruments are presented in **Table 5**. Parameter estimates from the PASE and SEEB CFA models are reported in the Supplementary Material.

**Table 5:** Simultaneous cross-group and longitudinal invariance results.

Model	χ^2^ (*df)*	*p*	χ^2^_diff_	*p*	Δ*df*	RMSEA [90% CI]	CFI	ΔCFI

PASE								
M1	151.412 (94)	<0.001	–	–	–	0.035 [0.024, 0.045]	0.992	–
M2	186.943 (116)	<0.001	39.034	<0.001	22	0.035 [0.025, 0.044]	0.990	-0.002
M3	170.224 (112)	<0.001	24.308	0.145	18	0.032 [0.022, 0.041]	0.991	0.001

**SEEB^a^**							

M1	115.659 (58)	<0.001	–	–	–	0.044 [0.032, 0.056]	0.987	–
M2	163.702 (76)	<0.001	48.136	<0.001	18	0.048 [0.038, 0.058]	0.981	-0.006


*χ^*2*^_diff_, adjusted χ^*2*^ difference test; *df*, degrees of freedom; PASE, Physical Activity Self-Efficacy Scale; SEEB, Self-Efficacy for Exercise Behaviors Scale; RMSEA, root mean square error of approximation; 90% CI, 90% confidence interval for RMSEA; CFI, comparative fit index; *Δ*CFI, change in comparative fit index; M1, configural model; M2, metric model; M3, partial metric model. ^a^SEEB model results from modified 5-item scale without items 2 and 3.*

##### Physical Activity Self-Efficacy Scale (PASE)

The configural model demonstrated adequate fit based on alternative fit indices (χ^2^ = 151.412, *df* = 94, *p* < 0.001, RMSEA = 0.035, CFI = 0.992). Given the large chi-square value, we examined the residual correlations and MIs for potential problems. All of the residual correlations for the intervention group were <0.10. For the control group, residual correlations ≥0.10 were the same as those reported for the longitudinal configural model. Metric model results did not support invariant factor loadings across group and time simultaneously (χ^2^ = 186.943, *df* = 116, *p* < 0.001, Δχ^2^ = 39.034, Δ*df* = 22, *p* = 0.014, RMSEA = 0.035, CFI = 0.990, ΔCFI = -0.002).

Although a partial metric invariance solution was considered unacceptable for this study, we attempted to identify if there was a single non-invariant factor loading by freeing each loading one at a time. This procedure showed that when item 6, “*I can be active in my free time on most days*,” was freed, partial metric invariance was met (χ^2^ = 170.224, *df* = 112, *p* < 0.001, Δχ^2^ = 24.308, Δ*df* = 18, *p* = 0.145, RMSEA = 0.032, CFI = 0.991, ΔCFI = -0.001). For this model, all of the residual correlations for the intervention group were <0.10. In the control group, four residual correlations ≥0.10 were found: (1) item 1 at baseline with item 6 at baseline (-0.100); (2) item 6 at baseline with item 3 at post-intervention (0.114); (3) item 3 at post-intervention with item 4 at post-intervention; and (4) item 3 at post-intervention with item 6 at post-intervention.

##### Self-Efficacy for Exercise Behaviors Scale (SEEB)

The configural model demonstrated adequate fit based on alternative fit indices (χ^2^ = 138.445, *df* = 66, *p* < 0.001, RMSEA = 0.047, CFI = 0.984). Areas of strain included a residual correlation of -0.102 between item 4 at baseline and item 5 at post-intervention in the control group and a residual correlation of 0.106 between item 7 at baseline and item 6 at post-intervention. All other residual correlations were <0.10. Similar to the PASE, the metric model indicated that factor loadings varied across group and time simultaneously (χ^2^ = 163.702, *df* = 76, *p* < 0.001, Δχ^2^ = 48.136, Δ*df* = 18, *p* < 0.001, RMSEA = 0.048, CFI = 0.981, ΔCFI = -0.006). We assessed each item, but found that achieving partial metric invariance would require freeing multiple non-invariant factor loadings.

### Reliability

#### Physical Activity Self-Efficacy Scale (PASE)

Reliability for the PASE invariance models ranged from 0.772 to 0.842. For the control group, reliability was quite consistent from baseline to post-intervention with minor differences in coefficients (Δ range: -0.001–0.003). On the other hand, the intervention group had larger longitudinal changes in reliability, all indicating increases over time (Δ range: 0.042–0.059).

#### Self-Efficacy for Exercise Behaviors Scale (SEEB)

For the SEEB invariance models, reliability coefficients ranged from 0.719 to 0.800. Similar to the PASE, the reliability coefficients were more stable in the control group than the intervention group over time. From baseline to post-intervention, changes in coefficients for the control group (Δ range: 0.009–0.018) were smaller than those for the intervention group (Δ range: 0.035–0.042). Reliability coefficients increased longitudinally. Overall, reliability estimates for the SEEB were slightly lower than those for the PASE. Reliability coefficients for invariance models are summarized in **Table 6**.

**Table 6:** Composite reliability estimates.

	Composite reliability estimates			
	
	Intervention group		Control group	
		
Instrument and model	Time 1	Time 2	Time 1	Time 2
**PASE**				
Cross group configural model	0.798	0.842	0.795	0.797
Cross group metric model	0.772	0.831	0.795	0.798
Cross group scalar model	0.793	0.840	0.795	0.798
Longitudinal configural model	0.797	0.836	0.795	0.798
Longitudinal metric model	0.797	0.842	0.795	0.794
Longitudinal scalar model	0.798	0.840	0.796	0.795
Simultaneous configural model	0.797	0.842	0.795	0.798
Simultaneous metric model	0.779	0.828	0.797	0.798
**SEEB**				
Cross group configural model	0.762	0.800	0.720	0.736
Cross group metric model	0.758	0.800	0.719	0.736
Cross group scalar model	0.761	0.799	0.719	0.736
Longitudinal configural model	0.761	0.800	0.719	0.737
Longitudinal metric model	0.761	0.799	0.719	0.732
Longitudinal scalar model	0.762	0.797	0.719	0.733
Simultaneous configural model	0.761	0.800	0.719	0.737
Simultaneous metric model	0.761	0.799	0.724	0.733


*Results computed using standardized factor loadings and thresholds; Time 1, baseline; Time 2, post-intervention; PASE, Physical Activity Self-Efficacy Scale; SEEB, Self-Efficacy for Exercise Behaviors Scale.*

## Discussion

We investigated the factorial validity, cross-group and longitudinal invariance, and composite reliability of two PA self-efficacy instruments used in the “Girls on the Move” group RCT. Both instruments demonstrated configural invariance for simultaneous analysis of cross-group and longitudinal invariance based on alternative fit indices. However, simultaneous metric invariance was not met for the PASE or the SEEB instruments. Partial metric invariance for the simultaneous analysis was achieved for the PASE with one factor loading identified as non-invariant. Partial metric invariance was not met for the SEEB. Longitudinal scalar invariance was achieved for both instruments in the control group but not the intervention group. Reliability was more stable over time in the control group for both instruments and higher for the PASE than the SEEB. Our findings regarding the measurement of PA self-efficacy are important for advancing the science of adolescent PA research, particularly because invariance testing for psychosocial constructs proposed to influence PA is not routinely conducted. These findings also point out the importance of investigating measurement invariance prior to making mean comparisons between groups for constructs included in intervention studies.

The PASE single factor model supported the hypothesized unidimensional factor structure. These findings are similar to those reported by earlier researchers with an 8-item instrument (Motl et al., 2000; Dishman et al., 2002, 2010). In addition, Steele et al. (2013) reported a unidimensional factor structure with the same 6-item instrument used in the current study. The fact that the intervention group demonstrated better fit at both time periods than the control group was not anticipated as comparability of groups should be expected in a group RCT. While significant differences emerged in the racial composition between groups, with significantly more black girls in the control group, Dishman et al. (2010) reported racial invariance for the 8-item version of this instrument with black and white girls of similar age.

The hypothesized SEEB factor structure did not fit the data well and required several model re-specifications, including deletion of two items. This instrument was first created and tested among adults resulting in a 12-item two-factor instrument, including a 5-item resisting-relapse factor and a 7-item making-time-for-exercise factor (Sallis et al., 1988). In subsequent psychometric studies that adapted the current SEEB, researchers mention the use of a resisting-relapse factor, but utilized items from both factors to measure PA self-efficacy among adolescents (Lawman et al., 2011; Peterson et al., 2013).

The PASE exhibited equal factor loadings between intervention and control groups at baseline and post-intervention but only partial scalar invariance. Longitudinal metric invariance was achieved for both groups separately with the control group also demonstrating scalar invariance. However, the factor loadings were not fully invariant when we simultaneously tested invariance across groups and time, indicating non-equivalent measurement of PA self-efficacy.

Partial metric invariance was investigated for the PASE for the simultaneous invariance analysis with one item found to be non-invariant: “*I can be active in my free time on most days*.” This finding has important measurement implications for the concept because deleting this item may better reflect the conceptual definition of PA self-efficacy. This item is the only one that does not include overcoming a barrier to PA or having the needed skills to participate in PA, which are theorized to be two dimensions of the concept (Voskuil and Robbins, 2015). Bandura (2004) stresses that self-efficacy should be assessed in the context of the challenges related to completing a particular behavior in order to maintain conceptual precision.

The modified 5-item SEEB exhibited cross-group metric and scalar invariance at baseline. Post-intervention results demonstrated metric invariance but only partial scalar invariance. Factor loadings were invariant over time in both groups. The modified SEEB demonstrated complete scalar invariance for the control group but partial scalar invariance in the intervention group. Similar to the PASE, the factor loadings varied in the simultaneous analysis. Unlike the PASE, partial metric invariance was not met. Freeing a single loading was insufficient to achieve invariance, implying that at least two of the five items had non-invariant factor loadings.

While neither instrument achieved scalar invariance for the simultaneous analysis, both measures demonstrated longitudinal scalar invariance for the control group but only partial scalar invariance for the intervention group. Additionally, reliability coefficients demonstrated less stability over time in the intervention group compared to the control group. These findings, along with the absence of equal factor loadings for the simultaneous invariance analysis, offer support for the theory that the intervention itself may influence how girls respond to the self-efficacy items and imply that the same concept is not being measured in the same way across groups and time.

Evaluation of group differences for adolescent girls’ PA self-efficacy should be interpreted cautiously due to the possibility of confounding from non-equivalent measurement. For example, if self-efficacy mean scores for girls in the intervention group were significantly higher or lower compared to the control group, these differences could be related to systematic response bias rather than an intervention effect on self-efficacy. Likewise, any conclusions regarding the mediational effects of PA self-efficacy in the “Girls on the Move” intervention study may be misleading in the absence of equivalent measurement across groups and time.

Our results underscore the importance of assessing the psychometric properties of adapted instruments rather than assuming that revised versions will be equally reliable and valid as the original. The PASE was created by deleting two items that were closely related to social support for PA, specifically questions about parental support and friend support, from an existing 8-item PA self-efficacy instrument. Steele et al. (2013) reported lower factor loadings for these two items in a sample of 6th–8th grade youth: 0.267 for parental support; 0.444 for friend support. Dewar et al. (2013) point out that the original 8-item instrument also had some lower factor loadings in earlier psychometric studies. Motl et al. (2000) reported factor loadings ranging from 0.390 to 0.610 indicating the possibility that some items may have been weakly related to the self-efficacy latent construct.

The absence of simultaneous metric invariance for the PASE may have been a consequence of these changes. In the original psychometric development study, three factors were described: support seeking, barriers, and positive alternatives (Saunders et al., 1997). The 8-item version of this instrument was unidimensional when items from all three factors were included. Deleting the items of parental and friend support, which represented the support seeking factor, may have led to this lack of invariance. Reducing response burden for adolescent girls is certainly a worthy endeavor, but may jeopardize reliability and validity.

The SEEB may be more useful than the PASE for assessing a participant’s ability to actually adhere to a physical activity regimen in an ongoing intervention. An awareness of issues interfering with adherence at certain time points during an intervention may allow interventionists to tailor their approach to specifically address the identified needs. In contrast, when no structured PA program exists, such as during the pre- or post-intervention time periods, the PASE may be more appropriate and lead to increased accuracy of participants’ responses. Thus the use of the SEEB may have been a poor fit for girls in this study given that they were enrolled in the group RCT only if they did not meet national guidelines for PA. Asking girls questions about sticking to an exercise program if none exists may have contributed to the non-invariance of the instrument in this study. Items in the PASE specifically asked about PA which is not limited to a structured exercise regimen.

Interestingly, the SEEB items demonstrated more variability in responses and were less skewed compared to the PASE items. The PASE items resulted in ceiling effects with the majority of girls choosing *agree a lot* for most items. Bandura (2006) recommended increasing the difficulty level for endorsing items when the majority of the sample selects the highest efficacy category. For the PASE, how the item stem is phrased and what response choices are provided could use revision. Given that the SEEB achieved better distribution of responses using the wording *how sure are you*, changing the item stem for the PASE from *I can…* to *How sure are you that you can…* could be examined in a future study. Bandura’s (1997, p. 43) instruction to include ‘degrees of assurance’ using words such as how certain, how confident, or how sure when rating indicator items supports this recommendation. Additionally, having girls rate their PA self-efficacy on a scale of 1–10, as recommended by Bandura, may decrease ceiling effects.

### Strengths and Limitations

A major strength of this study was the simultaneous cross-group and longitudinal assessment of invariance of an intervention and control group from a large, group RCT. Very few studies have conducted this level of invariance analysis for self-efficacy instruments between intervention and control groups in adolescent PA intervention research.

An added strength was the use of an appropriate estimator for ordinal data (i.e., WLSMV estimation). A frequently encountered issue in psychometric studies has been the application of the maximum likelihood estimator to Likert-type scales, particularly when ≤4 response options are used to assess a latent construct (Flora and Curran, 2004). This approach leads to biased parameter estimates such as lower factor loadings and elevated standard errors compared to parameter estimates using WLSMV estimation or other appropriate estimators for handling non-normal data (Brown, 2015).

This study involved rigorous invariance testing using recent recommendations by Kline (2016) in which chi-square tests and chi-square difference testing were not simply disregarded as inflated based on sample size, but rather used as a guide to evaluate potential areas of strain in a model. Relying only on changes in CFI between models to establish levels of invariance may not provide sufficient evidence of measurement invariance. While Cheung and Rensvold (2002) recommend using CFI changes <-0.01 as confirmation of invariance, their simulation relied on normally distributed data and maximum likelihood estimation. Additional simulations based on WLSMV estimation are needed to confirm these recommendations when using ordinal indicators (Dimitrov, 2010; Brown, 2015).

A limitation of this study was our use of single regression imputation for missing data. Although overall the amount of missing data was small, this approach may have reduced variability in the data, particularly at post-intervention when missing data increased due to attrition. While this study contributes to understanding factorial validity of self-efficacy measures, it did not address other aspects of validity such as convergent, discriminant, and criterion-related validity. Another limitation is that the sample included only urban, adolescent girls, and findings cannot be generalized to other populations, such as boys. Finally, while partial invariance was assessed in this study, the process for doing so with WLSMV estimation has not been fully investigated and remains an ongoing issue in SEM research (Dimitrov, 2010; Brown, 2015).

## Conclusion

Our results provide important information regarding the factorial validity, measurement invariance, and reliability of two PA self-efficacy instruments. This study indicated that neither of the adapted instruments achieved full metric invariance implying that these instruments did not measure the same concept equally between groups at both time points. These findings offer some support for the notion that participation in a PA intervention changes girls’ perceptions about a psychological construct such as self-efficacy. Perhaps as girls participated in various components of the intervention (Robbins et al., 2013), their enhanced understanding of the challenges related to PA may have altered the meaning of the construct over time and changed how they responded to items.

Several implications for future research arise from this study. We encourage investigators to conduct invariance analysis when adapting instruments that have previously been confirmed as valid and reliable. Essentially an adapted measure is an entirely different measure, and these alterations can potentially undermine the psychometric properties of an instrument. Assessing simultaneous group and longitudinal invariance in intervention studies involving girls could help to clarify whether decreases in self-efficacy have actually occurred or can be attributed to measurement problems.

Qualitative research using focus groups of adolescent girls may be one way to revise items in PA self-efficacy instruments. This approach may help ensure that items accurately reflect current challenges to PA in this population, particularly given technological advances over the last decade. The items in the instruments used for this study did not ask about use of computers, cell phones, iPads, or other devices that may interfere with an adolescent’s capability to be physically active. Other researchers have pointed out this deficit and have revised items on instruments to reflect current technology use among adolescents (Dewar et al., 2013).

Adequate psychometric evaluation of scales used to measure psychosocial constructs, such as self-efficacy, has been identified as a significant gap in the literature (Brown et al., 2009). This study contributes to an increased understanding of the psychometric properties of PA self-efficacy instruments. Continued assessment of PA self-efficacy instruments will help ensure that researchers measure this concept appropriately, providing a solid foundation for the science of adolescent PA research. Effort in this area is critical for furthering understanding of the role of PA self-efficacy in health behavior and how the concept might influence or be influenced by interventions designed for adolescent girls.

## Ethics Statement

This study and the group randomized controlled trial were conducted in accordance with the recommendations of the Michigan State University Institutional Review Board (MSU IRB). In accordance with the Declaration of Helsinki, all parents/guardians and adolescents provided written informed consent and assent, respectively. The MSU IRB approved the protocol.

## Author Contributions

VV, SP, and LR contributed to the study conception and design. VV conducted the data analysis and drafted the manuscript. SP and LR edited and provided critical revisions to the manuscript. SP offered assistance with data analysis and interpretation. LR provided acquisition to the data.

## Conflict of Interest Statement

The authors declare that the research was conducted in the absence of any commercial or financial relationships that could be construed as a potential conflict of interest.

## Footnotes

1 www.weighandmeasure.com
