# Supplementary material for: Comparing the Psychometric Properties of Two Physical Activity Self-Efficacy Instruments in Urban, Adolescent Girls: Validity, Measurement Invariance, and Reliability
Source: Front Psychol. 2017 Aug 3;8:1301. doi: 10.3389/fpsyg.2017.01301 (PMC5540903; doi:10.3389/fpsyg.2017.01301)
Supplement: Supplementary file 1 [file Data_Sheet_1.PDF]

Supplementary Material

Comparing the Psychometric Properties of Two Physical Activity Self-Efficacy Instruments in Urban, Adolescent Girls: Validity, Measurement Invariance, and Reliability

Table A. PASE Items Pooled Across Groups

	Baseline						Post-Intervention					
Items	1	2	3	4	5	6	1	2	3	4	5	6
1	1.00						1.00					
2	.47	1.00					.59	1.00				
3	.41	.43	1.00				.55	.49	1.00			
4	.54	.58	.48	1.00			.63	.55	.47	1.00		
5	.49	.46	.47	.52	1.00		.52	.50	.51	.51	1.00	
6	.38	.43	.45	.49	.46	1.00	.50	.46	.52	.53	.46	1.00
Mean	2.29	2.34	2.17	2.32	2.20	1.86	2.26	2.32	2.18	2.25	2.23	1.82
SD	.78	.85	.85	.80	.80	.97	.76	.78	.84	.80	.78	.95
Variance	.61	.71	.72	.65	.64	.95	.58	.61	.71	.63	.61	.91
Skewness	-1.00	-1.18	-.81	-1.11	-.76	-.36	-.88	-.90	-.74	-.95	-.76	-.35
Kurtosis	3.65	3.64	2.97	3.77	3.01	2.06	3.54	3.10	2.81	3.56	3.05	2.17
RSP-0	35	48	49	42	34	99	31	23	43	42	28	106
RSP-1	97	104	146	92	143	261	102	130	62	102	136	252
RSP-2	419	317	400	383	423	331	457	363	394	437	432	377
RSP-3	461	543	417	495	412	321	422	496	413	431	416	277
ICC[a]	.000	.000	.009	.000	.009	.003	.000	.002	.000	.003	.000	.010
95% UB	.000	.000	.060	.000	.060	.260	.000	.810	.000	.210	.000	.060

Note. ICC=intra-class correlation coefficient; PASE=Physical Activity Self-Efficacy scale; RSP=response option with counts presented for each item (0=disagree a lot; 1=disagree a little; 2=agree a little; 3=agree a lot). SD=standard deviation. UB=upper bound for ICC confidence interval (lower bound was 0 for all items).

[a]Intra-class correlation coefficients were used to examine non-independence in the data due to clustered sampling design by schools.

Table B. PASE Items by Treatment Group

	Baseline						Post-Intervention					
Control	1	2	3	4	5	6	1	2	3	4	5	6
Item 1	1.00						1.00					
Item 2	.47	1.00					.55	1.00				
Item 3	.45	.41	1.00				.48	.45	1.00			
Item 4	.55	.58	.42	1.00			.63	.52	.36	1.00		
Item 5	.54	.51	.51	.55	1.00		.49	.47	.47	.46	1.00	
Item 6	.34	.35	.46	.45	.49	1.00	.44	.42	.51	.45	.43	1.00
Mean	2.31	2.35	2.17	2.36	2.25	1.93	2.26	2.40	2.16	2.30	2.23	1.89
SD	.81	.82	.83	.80	.80	.95	.78	.76	.82	.76	.76	.94
Variance	.65	.68	.69	.63	.64	.90	.60	.58	.67	.58	.57	.88
Skewness	-1.15	-1.22	-.83	-1.20	-.81	-.47	-.93	-1.11	-.65	-.95	-.71	-.48
Kurtosis	3.94	3.90	3.18	4.01	2.94	2.23	3.54	3.60	2.69	3.54	3.01	2.35
Intervention	1	2	3	4	5	6	1	2	3	4	5	6
Item 1	1.00						1.00					
Item 2	.48	1.00					.62	1.00				
Item 3	.38	.46	1.00				.62	.53	1.00			
Item 4	.53	.59	.54	1.00			.63	.58	.56	1.00		
Item 5	.44	.40	.43	.48	1.00		.56	.52	.54	.55	1.00	
Item 6	.43	.50	.44	.53	.42	1.00	.55	.48	.54	.58	.48	1.00
Mean	2.27	2.32	2.17	2.27	2.15	1.80	2.25	2.23	2.17	2.18	2.21	1.75
SD	.75	.87	.87	.81	.80	.99	.74	.79	.87	.83	.80	.97
Variance	.56	.75	.76	.66	.64	.99	.55	.63	.76	.69	.64	.93
Skewness	-.82	-1.13	-.78	-1.02	-.73	-.26	-.83	-.72	-.81	-.94	-.79	-.23
Kurtosis	3.29	3.41	2.78	3.58	3.10	1.94	3.54	2.78	2.89	3.48	3.05	2.05

Note. PASE=Physical Activity Self-Efficacy scale; SD=standard deviation.

Table C. SEEB Items Pooled Across Groups

	Baseline							Post-Intervention						
Items	1	2	3	4	5	6	7	1	2	3	4	5	6	7
1	1.00							1.00						
2	.50	1.00						.54	1.00					
3	.36	.37	1.00					.36	.29	1.00				
4	.31	.38	.30	1.00				.43	.39	.38	1.00			
5	.34	.41	.36	.44	1.00			.42	.32	.35	.41	1.00		
6	.36	.37	.33	.49	.50	1.00		.41	.32	.37	.47	.57	1.00	
7	.38	.40	.32	.36	.49	.51	1.00	.43	.42	.36	.34	.53	.50	1.00
Mean	1.79	1.89	1.62	2.34	1.93	1.97	1.78	1.73	1.79	1.68	2.19	1.83	1.87	1.64
SD	.89	.94	1.05	.79	.90	.86	.92	.90	.92	.98	.80	.91	.86	.95
Variance	.80	.88	1.10	.62	.81	.73	.84	.80	.84	.96	.64	.82	.74	.89
Skewness	-.37	-.47	-.21	-1.05	-.51	-.45	-.28	-.32	-.41	-.22	-.78	-.40	-.37	-.20
Kurtosis	2.43	2.32	1.86	3.48	2.50	2.47	2.23	2.38	2.38	2.04	3.10	2.39	2.48	2.14
RSP-0	95	96	197	30	79	53	94	105	109	142	36	93	67	138
RSP-1	245	219	229	111	208	227	279	261	227	275	137	236	249	286
RSP-2	448	399	347	353	429	428	396	445	442	364	436	431	449	391
RSP-3	224	298	239	518	296	304	243	201	234	231	403	252	247	197
ICC[a]	.001	.013	.000	.007	.000	.010	.008	.000	.004	.000	.008	.000	.000	.005
95% UB	.980	.060	.000	.070	.000	.060	.060	.000	.120	.000	.060	.000	.000	.090

Note. ICC=intra-class correlation coefficient; SEEB= Self-Efficacy for Exercise Behaviors scale; RSP=response option with counts presented for each item (0=disagree a lot; 1=disagree a little; 2=agree a little; 3=agree a lot); SD=standard deviation. UB=upper bound for ICC confidence interval (lower bound was 0 for all items).

[a]Intra-class correlation coefficients were used to examine non-independence in the data due to clustered sampling design by schools.

Table D. SEEB Items by Treatment Group

	Baseline							Post-Intervention						
Control	1	2	3	4	5	6	7	1	2	3	4	5	6	7
Item 1	1.00							1.00						
Item 2	.44	1.00						.50	1.00					
Item 3	.36	.37	1.00					.36	.23	1.00				
Item 4	.30	.34	.27	1.00				.47	.38	.36	1.00			
Item 5	.31	.39	.34	.44	1.00			.37	.26	.38	.37	1.00		
Item 6	.35	.25	.30	.48	.45	1.00		.37	.25	.37	.44	.52	1.00	
Item 7	.33	.37	.33	.35	.46	.47	1.00	.39	.38	.36	.32	.42	.44	1.00
Mean	1.78	1.91	1.62	2.33	1.96	2.05	1.82	1.73	1.77	1.68	2.25	1.79	1.88	1.61
SD	.91	.92	1.08	.78	.88	.81	.92	.91	.94	1.01	.79	.94	.85	.95
Variance	.83	.84	1.16	.61	.77	.66	.84	.83	.88	1.01	.62	.88	.73	.91
Skewness	-.42	-.50	-.23	-1.00	-.52	-.41	-.32	-.32	-.41	-.23	-.84	-.34	-.39	-.14
Kurtosis	2.43	2.44	1.80	3.41	2.55	2.39	2.23	2.33	2.33	1.98	3.15	2.22	2.53	2.10
Intervention	P7-1	P7-2	P7-3	P7-4	P7-5	P7-6	P7-7	P7-1	P7-2	P7-3	P7-4	P7-5	P7-6	P7-7
P7-1	1.00							1.00						
P7-2	.56	1.00						.58	1.00					
P7-3	.36	.37	1.00					.35	.35	1.00				
P7-4	.32	.42	.34	1.00				.40	.40	.40	1.00			
P7-5	.36	.42	.37	.45	1.00			.48	.39	.32	.46	1.00		
P7-6	.38	.47	.37	.50	.54	1.00		.44	.38	.37	.50	.63	1.00	
P7-7	.42	.42	.32	.37	.52	.54	1.00	.48	.47	.36	.37	.64	.56	1.00
Mean	1.80	1.87	1.62	2.36	1.91	1.89	1.74	1.74	1.81	1.68	2.13	1.87	1.85	1.67
SD	.88	.96	1.02	.80	.92	.89	.91	.88	.90	.95	.81	.87	.87	.94
Variance	.77	.92	1.04	.63	.84	.80	.83	.77	.81	.91	.65	.76	.75	.88
Skewness	-.32	-.44	-.19	-1.10	-.50	-.43	-.24	-.32	-.40	-.20	-.72	-.46	-.34	-.25
Kurtosis	2.41	2.22	1.92	3.56	2.44	2.42	2.23	2.43	2.43	2.11	3.07	2.57	2.43	2.20

Note. SEEB= Self-Efficacy for Exercise Behaviors scale; SD=standard deviation.

Table E. PASE Simultaneous Cross-Group and Longitudinal Invariance: Configural Model Estimates

Group/Item	Baseline			Post-Intervention		
Intervention	λ (SE)	Std. λ (SE)	R^2 (SE)	λ (SE)	Std. λ (SE)	R^2 (SE)
Item 1	1.006 (.067)	.671 (.035)	.450 (.047)	1.166 (.052)	.819 (.024)	.670 (.039)
Item 2	1.074 (.071)	.716 (.032)	.513 (.046)	1.033 (.050)	.726 (.027)	.526 (.039)
Item 3	.973 (.065)	.649 (.034)	.421 (.044)	1.065 (.048)	.748 (.025)	.559 (.038)
Item 4	1.205 (.069)	.804 (.028)	.646 (.045)	1.123 (.047)	.789 (.023)	.622 (.036)
Item 5	.930 (.072)	.626 (.028)	.392 (.046)	1.005 (.053)	.706 (.029)	.498 (.041)
Item 6	1.000	.667 (.033)	.445 (.044)	1.000	.702 (.027)	.493 (.038)
	τ_1 (SE)	τ_2 (SE)	τ_3 (SE)	τ_1 (SE)	τ_2 (SE)	τ_3 (SE)
Item 1	-1.986 (.121)	-1.102 (.070)	.183 (.056)	-1.920 (.114)	-1.139 (.071)	.248 (.056)
Item 2	-1.654 (.094)	-.967 (.066)	-.103 (.056)	-1.986 (.121)	-.929 (.065)	.168 (.056)
Item 3	-1.654 (.094)	-.807 (.063)	.178 (.056)	-1.617 (.092)	-.835 (.063)	.198 (.056)
Item 4	-1.715 (.098)	-1.066 (.069)	.098 (.056)	-1.599 (.091)	-1.007 (.067)	.264 (.056)
Item 5	-1.760 (.101)	-.921 (.065)	.346 (.057)	-1.834 (.107)	-.952 (.066)	.213 (.056)
Item 6	-1.238 (.074)	-.264 (.056)	.508 (.058)	-1.207 (.073)	-.258 (.056)	.653 (.060)
Control	λ (SE)	Std. λ (SE)	R^2 (SE)	λ (SE)	Std. λ (SE)	R^2 (SE)
Item 1	1.134 (.084)	.689 (.034)	.474 (.047)	1.190 (.068)	.772 (.029)	.597 (.045)
Item 2	1.131 (.079)	.686 (.033)	.471 (.046)	1.044 (.066)	.678 (.034)	.459 (.046)
Item 3	1.053 (.077)	.639 (.035)	.409 (.045)	1.030 (.064)	.669 (.033)	.447 (.044)
Item 4	1.266 (.084)	.768 (.030)	.590 (.046)	1.100 (.065)	.714 (.030)	.510 (.043)
Item 5	1.253 (.079)	.761 (.027)	.579 (.042)	1.045 (.062)	.678 (.033)	.460 (.045)
Item 6	1.000	.607 (.035)	.368 (.042)	1.000	.649 (.031)	.422 (.040)
	τ_1 (SE)	τ_2 (SE)	τ_3 (SE)	τ_1 (SE)	τ_2 (SE)	τ_3 (SE)
Item 1	-1.687 (.097)	-1.148 (.072)	.040 (.056)	-1.827 (.107)	-1.101 (.070)	.171 (.056)
Item 2	-1.687 (.097)	-1.110 (.070)	-.080 (.056)	-2.016 (.125)	-1.148 (.072)	-.120 (.056)
Item 3	-1.666 (.096)	-.933 (.066)	.268 (.057)	-1.854 (.110)	-.830 (.064)	.268 (.057)
Item 4	-1.753 (.102)	-1.167 (.072)	-.045 (.056)	-1.913 (.115)	-1.138 (.071)	.110 (.056)
Item 5	-1.913 (.115)	-.949 (.066)	.125 (.056)	-2.016 (.125)	-1.022 (.068)	.237 (.057)
Item 6	-1.355 (.079)	-.482 (.058)	.444 (.058)	-1.307 (.077)	-.499 (.059)	.551 (.059)

Note. λ =factor loading; τ =unstandardized threshold; PASE=Physical Activity Self-Efficacy scale; Std=standardized ; SE=standard error.

Table F. PASE Simultaneous Cross-Group and Longitudinal Invariance: Metric Model Estimates

Group/Item	Baseline			Post-Intervention		
Intervention	λ (SE)	Std. λ (SE)	R^2 (SE)	λ (SE)	Std. λ (SE)	R^2 (SE)
Item 1	1.132 (.047)	.699 (.029)	.489 (.041)	1.132 (.047)	.820 (.022)	.673 (.036)
Item 2	1.078 (.046)	.692 (.027)	.479 (.037)	1.078 (.046)	.740 (.025)	.548 (.037)
Item 3	1.045 (.043)	.654 (.028)	.428 (.037)	1.045 (.043)	.737 (.023)	.543 (.034)
Item 4	1.183 (.047)	.796 (.025)	.633 (.040)	1.183 (.047)	.785 (.021)	.616 (.033)
Item 5	1.131 (.046)	.631 (.030)	.398 (.038)	1.131 (.046)	.708 (.027)	.501 (.038)
Item 6	1.000	.665 (.028)	.442 (.038)	1.000	.699 (.025)	.489 (.035)
	τ_1 (SE)	τ_2 (SE)	τ_3 (SE)	τ_1 (SE)	τ_2 (SE)	τ_3 (SE)
Item 1	-1.850 (.068)	-1.287 (.084)	-.169 (.105)	-1.850 (.068)	-1.235 (.075)	-.136 (.104)
Item 2	-1.807 (.073)	-1.123 (.073)	-.399 (.087)	-1.807 (.073)	-1.094 (.077)	-.177 (.100)
Item 3	-1.699 (.067)	-.996 (.074)	-.150 (.099)	-1.699 (.067)	-.987 (.068)	-.146 (.098)
Item 4	-1.758 (.067)	-1.196 (.073)	-.264 (.102)	-1.758 (.067)	-1.220 (.074)	-.120 (.111)
Item 5	-1.996 (.081)	-1.216 (.087)	.006 (.116)	-1.996 (.081)	-1.206 (.081)	-.137 (.108)
Item 6	-1.304 (.054)	-.264 (.056)	.508 (.058)	-1.304 (.054)	-.499 (.048)	.242 (.116)
Control	λ (SE)	Std. λ (SE)	R^2 (SE)	λ (SE)	Std. λ (SE)	R^2 (SE)
Item 1	1.132 (.047)	.717 (.024)	.514 (.035)	1.132 (.047)	.718 (.023)	.515 (.033)
Item 2	1.078 (.046)	.683 (.024)	.467 (.033)	1.078 (.046)	.683 (.025)	.467 (.034)
Item 3	1.045 (.043)	.662 (.025)	.438 (.033)	1.045 (.043)	.662 (.024)	.438 (.032)
Item 4	1.183 (.047)	.750 (.022)	.562 (.034)	1.183 (.047)	.750 (.024)	.562 (.035)
Item 5	1.131 (.046)	.716 (.024)	.513 (.035)	1.131 (.046)	.716 (.025)	.513 (.036)
Item 6	1.000	.633 (.025)	.401 (.032)	1.000	.634 (.025)	.402 (.032)
	τ_1 (SE)	τ_2 (SE)	τ_3 (SE)	τ_1 (SE)	τ_2 (SE)	τ_3 (SE)
Item 1	-1.850 (.068)	-1.148 (.072)	.040 (.056)	-1.850 (.068)	-1.101 (.070)	.171 (.056)
Item 2	-1.807 (.073)	-1.110 (.070)	-.080 (.056)	-1.807 (.073)	-1.148 (.072)	-.120 (.056)
Item 3	-1.699 (.067)	-.933 (.066)	.268 (.057)	-1.699 (.067)	-.830 (.064)	.268 (.057)
Item 4	-1.758 (.067)	-1.167 (.072)	-.045 (.056)	-1.758 (.067)	-1.138 (.071)	.110 (.056)
Item 5	-1.996 (.081)	-.949 (.066)	.125 (.056)	-1.996 (.081)	-1.022 (.068)	.237 (.057)
Item 6	-1.304 (.054)	-.499 (.048)	.444 (.058)	-1.304 (.054)	-.499 (.048)	.551 (.059)

Note. λ =factor loading; τ =unstandardized threshold; PASE=Physical Activity Self-Efficacy scale; Std=standardized ; SE=standard error.

Table G. SEEB Simultaneous Cross-Group and Longitudinal Invariance: Configural Model Estimates

Group/Item		Baseline			Post-Intervention	
Intervention	λ (SE)	Std. λ (SE)	R^2 (SE)	λ (SE)	Std. λ (SE)	R^2 (SE)
Item 1	.747 (.061)	.520 (.037)	.271 (.039)	.817 (.053)	.613 (.034)	.376 (.042)
Item 4	.924 (.069)	.644 (.040)	.414 (.051)	.775 (.054)	.582 (.035)	.339 (.041)
Item 5	.999 (.064)	.696 (.032)	.484 (.045)	1.099 (.046)	.826 (.023)	.682 (.037)
Item 6	1.125 (.064)	.783 (.027)	.614 (.043)	1.021 (.047)	.767 (.025)	.588 (.039)
Item 7	1.000	.697 (.031)	.485 (.044)	1.000	.751 (.027)	.564 (.040)
	τ_1 (SE)	τ_2 (SE)	τ_3 (SE)	τ_1 (SE)	τ_2 (SE)	τ_3 (SE)
Item 1	-1.402 (.081)	-.404 (.057)	.767 (.062)	-1.304 (.077)	-.362 (.057)	.863 (.064)
Item 4	-1.861 (.109)	-1.084 (.069)	-.069 (.056)	-1.737 (.100)	-.899 (.064)	.367 (.057)
Item 5	-1.352 (.078)	-.541 (.059)	.559 (.059)	-1.416 (.081)	-.536 (.058)	.690 (.061)
Item 6	-1.429 (.082)	-.508 (.058)	.599 (.059)	-1.486 (.085)	-.458 (.058)	.703 (.061)
Item 7	-1.293 (.076)	-.294 (.056)	.767 (.062)	-1.129 (.070)	-.258 (.056)	.849 (.063)
Control	λ (SE)	Std. λ (SE)	R^2 (SE)	λ (SE)	Std. λ (SE)	R^2 (SE)
Item 1	.731 (.068)	.491 (.040)	.241 (.040)	.980 (.081)	.612 (.037)	.374 (.046)
Item 4	.902 (.069)	.606 (.038)	.367 (.046)	1.033 (.083)	.645 (.039)	.416 (.050)
Item 5	.980 (.075)	.658 (.037)	.433 (.049)	1.053 (.085)	.658 (.039)	.432 (.051)
Item 6	1.085 (.076)	.728 (.034)	.530 (.049).	1.103 (.082)	.689 (.034)	.474 (.047)
Item 7	1.000	.671 (.034)	.451 (.045)	1.000	.624 (.035)	.390 (.044)
	τ_1 (SE)	τ_2 (SE)	τ_3 (SE)	τ_1 (SE)	τ_2 (SE)	τ_3 (SE)
Item 1	-1.240 (.075)	-.444 (.058)	.768 (.062)	-1.218 (.074)	-.346 (.057)	.830 (.064)
Item 4	-1.913 (.115)	-1.083 (.070)	.010 (.056)	-1.883 (.112)	-1.005 (.068)	.150 (.056)
Item 5	-1.493 (.086)	-.604 (.060)	.534 (.059)	-1.250 (.075)	-.373 (.057)	.665 (.061)
Item 6	-1.913 (.115)	-.684 (.061)	.449 (.058)	-1.524 (.087)	-.522 (.059)	.684 (.061)
Item 7	-1.355 (.079)	-.378 (.057)	.647 (.060)	-1.065 (.069)	-.150 (.056)	.873 (.064)

Note. λ =factor loading; τ =unstandardized threshold; SEEB= Self-Efficacy for Exercise Behaviors scale; Std=standardized ; SE=standard error.

Table H. SEEB Simultaneous Cross-Group and Longitudinal Invariance: Metric Model Estimates

Group/Item	Baseline			Post-Intervention		
Intervention	λ (SE)	Std. λ (SE)	R^2 (SE)	λ (SE)	Std. λ (SE)	R^2 (SE)
Item 1	.822 (.043)	.556 (.030)	.310 (.034)	.822 (.043)	.615 (.029)	.379 (.036)
Item 4	.952 (.045)	.638 (.032)	.407 (.040)	.952 (.045)	.598 (.029)	.358 (.035)
Item 5	1.024 (.050)	.698 (.027)	.487 (.038)	1.024 (.050)	.820 (.021)	.672 (.034)
Item 6	1.124 (.050)	.745 (.023)	.555 (.035)	1.124 (.050)	.770 (.022)	.592 (.034)
Item 7	1.000	.713 (.027)	.508 (.038)	1.000	.743 (.024)	.551 (.036)
	τ_1 (SE)	τ_2 (SE)	τ_3 (SE)	τ_1 (SE)	τ_2 (SE)	τ_3 (SE)
Item 1	-1.282 (.050)	-.396 (.068)	.791 (.132)	-1.282 (.050)	-.380 (.068)	.796 (.129)
Item 4	-1.927 (.076)	-1.094 (.086)	-.055 (.090)	-1.927 (.076)	-1.066 (.089)	.382 (.114)
Item 5	-1.340 (.053)	-.528 (.073)	.579 (.127)	-1.340 (.053)	-.522 (.067)	.579 (.126)
Item 6	-1.590 (.065)	-.507 (.080)	.639 (.140)	-1.590 (.065)	-.525 (.082)	.693 (.145)
Item 7	-1.183 (.051)	-.270 (.046)	.754 (.137)	-1.183 (.051)	-.270 (.046)	.781 (.139)
Control	λ (SE)	Std. λ (SE)	R^2 (SE)	λ (SE)	Std. λ (SE)	R^2 (SE)
Item 1	.822 (.043)	.528 (.026)	.279 (.028)	.822 (.043)	.535 (.025)	.286 (.027)
Item 4	.952 (.045)	.612 (.026)	.374 (.032)	.952 (.045)	.619 (.027)	.384 (.033)
Item 5	1.024 (.050)	.658 (.026)	.433 (.034)	1.024 (.050)	.666 (.026)	.444 (.035)
Item 6	1.124 (.050)	.723 (.026)	.522 (.037).	1.124 (.050)	.731 (.026)	.535 (.038)
Item 7	1.000	.643 (.026)	.413 (.033)	1.000	.651 (.025)	.423 (.033)
	τ_1 (SE)	τ_2 (SE)	τ_3 (SE)	τ_1 (SE)	τ_2 (SE)	τ_3 (SE)
Item 1	-1.282 (.050)	-.444 (.058)	.768 (.062)	-1.282 (.050)	-.346 (.057)	.830 (.064)
Item 4	-1.927 (.076)	-1.083 (.070)	.010 (.056)	-1.927 (.076)	-1.005 (.068)	.150 (.056)
Item 5	-1.340 (.053)	-.604 (.060)	.534 (.059)	-1.340 (.053)	-.373 (.057)	.665 (.061)
Item 6	-1.590 (.065)	-.684 (.061)	.449 (.058)	-1.590 (.065)	-.522 (.059)	.684 (.061)
Item 7	-1.183 (.051)	-.270 (.046)	.647 (.060)	-1.183 (.051)	-.270 (.046)	.873 (.064)

Note. λ =factor loading; τ =unstandardized threshold; SEEB= Self-Efficacy for Exercise Behaviors scale; Std=standardized ; SE=standard error.

SIMULATENOUS MEASUREMENT INVARIANCE – MPLUS SYNTAX

Note. In the Mplus syntax the PASE was referred to as PASE-6 and SEEB was referred to as PASE-7.

PASE-6 SIMULTANEOUS CONFIGURAL MODEL

```
TITLE: PASE-6 CONFIGURAL MODEL

DATA: FILE = PASE.MPLUS.dat;

VARIABLE:
NAMES = W0EFM W0EFTV W0EFH W0EFHM W0EFS W0EFB
    W0EFD W0EFC W0EFL W0EFEX W0EFHO W0EFPR W0EFSD
    W17ARM W17EFM W17EFTV W17EFH W17EFHM W17EFS W17EFB
    W17EFD W17EFC W17EFL W17EFEX W17EFHO W17EFPR W17EFSD;
USEVARIABLES = W0EFM W0EFTV W0EFH W0EFHM W0EFS W0EFB
        W17EFM W17EFTV W17EFH W17EFHM W17EFS W17EFB;
GROUPING = W17ARM (0=CONTROL 1=INTERVENTION);
CATEGORICAL = W0EFM W0EFTV W0EFH W0EFHM W0EFS W0EFB
        W17EFM W17EFTV W17EFH W17EFHM W17EFS W17EFB;

ANALYSIS:
ESTIMATOR = WLSMV;
PARAMETERIZATION = DELTA;

MODEL:
!Factor loadings free except first indicator
PASE6_T1 BY W0EFB W0EFM* W0EFTV* W0EFH* W0EFHM* W0EFS*;
PASE6_T2 BY W17EFB W17EFM* W17EFTV* W17EFH* W17EFHM* W17EFS*;
!Thresholds free
[W0EFB$1*];
[W0EFB$2*];
[W0EFB$3*];
[W0EFM$1*];
[W0EFM$2*];
[W0EFM$3*];
[W0EFTV$1*];
[W0EFTV$2*];
[W0EFTV$3*];
[W0EFH$1*];
[W0EFH$2*];
[W0EFH$3*];
[W0EFHM$1*];
[W0EFHM$2*];
[W0EFHM$3*];
[W0EFS$1*];
[W0EFS$2*];
[W0EFS$3*]
[W17EFB$1*];
[W17EFB$2*];
[W17EFB$3*];
[W17EFM$1*];
[W17EFM$2*];
[W17EFM$3*];
```

```
[W17EFTV$1*];
[W17EFTV$2*];
[W17EFTV$3*];
[W17EFH$1*];
[W17EFH$2*];
[W17EFH$3*];
[W17EFHM$1*];
[W17EFHM$2*];
[W17EFHM$3*];
[W17EFS$1*];
[W17EFS$2*];
[W17EFS$3*];
!Scale factors set to 1 in control group
{W0EFM-W0EFB@1 W17EFM-W17EFB@1};
!Factor variances and covariances all free
PASE6_T1* PASE6_T2*;
PASE6_T1 WITH PASE6_T2*;
!Factor means fixed at 0 in control group
[PASE6_T1@0 PASE6_T2@0];
!Error covariances for same indicator over time
W0EFM WITH W17EFM;
W0EFTV WITH W17EFTV;
W0EFH WITH W17EFH;
W0EFHM WITH W17EFHM;
W0EFS WITH W17EFS;
W0EFB WITH W17EFB;

MODEL INTERVENTION:
!Factor loadings free except first indicator
PASE6_T1 BY W0EFB@1 W0EFM* W0EFTV* W0EFH* W0EFHM* W0EFS*;
PASE6_T2 BY W17EFB@1 W17EFM* W17EFTV* W17EFH* W17EFHM* W17EFS*;
!Thresholds free
[W0EFB$1*];
[W0EFB$2*];
[W0EFB$3*];
[W0EFM$1*];
[W0EFM$2*];
[W0EFM$3*];
[W0EFTV$1*];
[W0EFTV$2*];
[W0EFTV$3*];
[W0EFH$1*];
[W0EFH$2*];
[W0EFH$3*];
[W0EFHM$1*];
[W0EFHM$2*];
[W0EFHM$3*];
[W0EFS$1*];
[W0EFS$2*];
[W0EFS$3*]
[W17EFB$1*];
[W17EFB$2*];
[W17EFB$3*];
[W17EFM$1*];
[W17EFM$2*];
[W17EFM$3*];
```

```
[W17EFTV$1*];
[W17EFTV$2*];
[W17EFTV$3*];
[W17EFH$1*];
[W17EFH$2*];
[W17EFH$3*];
[W17EFHM$1*];
[W17EFHM$2*];
[W17EFHM$3*];
[W17EFS$1*];
[W17EFS$2*];
[W17EFS$3*];
!Scale factors set to 1 in intervention group
{W0EFM-W0EFB@1 W17EFM-W17EFB@1};
!Factor variances and covariances all free
PASE6_T1* PASE6_T2*;
PASE6_T1 WITH PASE6_T2*;
!Factor means fixed at 0 in intervention group
[PASE6_T1@0 PASE6_T2@0];
!Error covariances for same indicator
W0EFM WITH W17EFM;
W0EFTV WITH W17EFTV;
W0EFH WITH W17EFH;
W0EFHM WITH W17EFHM;
W0EFS WITH W17EFS;
W0EFB WITH W17EFB;

OUTPUT:
SAMPSTAT;
STANDARDIZED (STDYX);
CINTERVAL;
RESIDUAL;
MODINDICES (10);
TECH1;
TECH5;

SAVEDATA:
DIFFTEST = PASE6_LI_MI_CONF_DERIVD.DAT;
```

PASE-6 SIMULTANEOUS METRIC MODEL

```
TITLE: PASE-6 SIMULATENOUS METRIC MODEL

DATA: FILE = PASE.MPLUS.dat;

VARIABLE:
NAMES = W0EFM W0EFTV W0EFH W0EFHM W0EFS W0EFB
      W0EFD W0EFC W0EFL W0EFEX W0EFHO W0EFPR W0EFSD
      W17ARM W17EFM W17EFTV W17EFH W17EFHM W17EFS W17EFB
      W17EFD W17EFC W17EFL W17EFEX W17EFHO W17EFPR W17EFSD;
USEVARIABLES = W0EFM W0EFTV W0EFH W0EFHM W0EFS W0EFB
            W17EFM W17EFTV W17EFH W17EFHM W17EFS W17EFB;
GROUPING = W17ARM (0=CONTROL 1=INTERVENTION);
CATEGORICAL =   W0EFM W0EFTV W0EFH W0EFHM W0EFS W0EFB
            W17EFM W17EFTV W17EFH W17EFHM W17EFS W17EFB;

ANALYSIS:
ESTIMATOR = WLSMV;
PARAMETERIZATION = DELTA;
DIFFTEST = PASE6_LI_MI_CONF_DERIVD.DAT;

MODEL:
!Factor loadings constrained to be equal at both time points
PASE6_T1 BY W0EFB !Fixed at one - referrent indicator
        W0EFM-W0EFS (1-5);
PASE6_T2 BY W17EFB !Fixed at one - referent indicator
        W17EFM-W17EFS (1-5);
!Thresholds free except those stated in Mplus user guide
[W0EFB$1*] (6); !First threshold of each item held equal
[W0EFB$2*] (7); !Second threshold of referent held equal
[W0EFB$3*];
[W0EFM$1*] (9);
[W0EFM$2*];
[W0EFM$3*];
[W0EFTV$1*] (12);
[W0EFTV$2*];
[W0EFTV$3*];
[W0EFH$1*] (15);
[W0EFH$2*];
[W0EFH$3*];
[W0EFHM$1*] (18);
[W0EFHM$2*];
[W0EFHM$3*];
[W0EFS$1*] (21);
[W0EFS$2*];
[W0EFS$3*];
[W17EFB$1*] (6);
[W17EFB$2*] (7);
[W17EFB$3*];
[W17EFM$1*] (9);
[W17EFM$2*];
[W17EFM$3*];
[W17EFTV$1*] (12);
[W17EFTV$2*];
[W17EFTV$3*];
```

```
[W17EFH$1*] (15);
[W17EFH$2*];
[W17EFH$3*];
[W17EFHM$1*] (18);
[W17EFHM$2*];
[W17EFHM$3*];
[W17EFS$1*] (21);
[W17EFS$2*];
[W17EFS$3*];
!Scale factors set to 1 in control group
{W0EFM-W0EFB@1 W17EFM-W17EFB@1};
!Factor variances and covariances all free
PASE6_T1* PASE6_T2*;
PASE6_T1 WITH PASE6_T2*;
!Factor means set at 0 in control group
[PASE6_T1@0 PASE6_T2@0];
!Error covariances for same indicator across time
W0EFM WITH W17EFM;
W0EFTV WITH W17EFTV;
W0EFH WITH W17EFH;
W0EFHM WITH W17EFHM;
W0EFS WITH W17EFS;
W0EFB WITH W17EFB;

MODEL INTERVENTION:
!Factor loadings constrained to be equal at both time points
PASE6_T1 BY W0EFB@1 !Fixed at one - referent indicator
        W0EFM-W0EFS (1-5);
PASE6_T2 BY W17EFB@1 !Fixed at one - referent indicator
        W17EFM-W17EFS (1-5);
!Thresholds free except those in directions in M+ user guide
[W0EFB$1*] (6); !First threshold of each item held equal
[W0EFB$2*] (7); !Second threshold of referent held equal
[W0EFB$3*];
[W0EFM$1*] (9);
[W0EFM$2*];
[W0EFM$3*];
[W0EFTV$1*] (12);
[W0EFTV$2*];
[W0EFTV$3*];
[W0EFH$1*] (15);
[W0EFH$2*];
[W0EFH$3*];
[W0EFHM$1*] (18);
[W0EFHM$2*];
[W0EFHM$3*];
[W0EFS$1*] (21);
[W0EFS$2*];
[W0EFS$3*];
[W17EFB$1*] (6);
[W17EFB$2*] (7);
[W17EFB$3*];
[W17EFM$1*] (9);
[W17EFM$2*];
[W17EFM$3*];
[W17EFTV$1*] (12);
```

```
[W17EFTV$2*];
[W17EFTV$3*];
[W17EFH$1*] (15);
[W17EFH$2*];
[W17EFH$3*];
[W17EFHM$1*] (18);
[W17EFHM$2*];
[W17EFHM$3*];
[W17EFS$1*] (21);
[W17EFS$2*];
[W17EFS$3*];
!Scale factors free in intervention group
{W0EFM-W0EFB W17EFM-W17EFB};
!Factor variances and covariances all free
PASE6_T1* PASE6_T2*;
PASE6_T1 WITH PASE6_T2*;
!Factor means set free in intervention group
[PASE6_T1 PASE6_T2];
!Error covariances for same indicator across time
W0EFM WITH W17EFM;
W0EFTV WITH W17EFTV;
W0EFH WITH W17EFH;
W0EFHM WITH W17EFHM;
W0EFS WITH W17EFS;
W0EFB WITH W17EFB;

OUTPUT:
SAMPSTAT;
STANDARDIZED (STDYX);
CINTERVAL;
RESIDUAL;
MODINDICES (10);
TECH1;
TECH5;

SAVEDATA:
DIFFTEST = PASE6_LI_MI_METRIC_DERIVD.DAT;
```

PASE-7 SIMULTANEOUS CONFIGURAL MODEL

```
TITLE: PASE-7 SIMULTANEOUS CONFIGURAL MODEL

DATA: FILE = PASE.MPLUS.dat;

VARIABLE:
NAMES = W0EFM W0EFTV W0EFH W0EFHM W0EFS W0EFB
       W0EFD W0EFC W0EFL W0EFEX W0EFHO W0EFPR W0EFSD
       W17ARM W17EFM W17EFTV W17EFH W17EFHM W17EFS W17EFB
       W17EFD W17EFC W17EFL W17EFEX W17EFHO W17EFPR W17EFSD;
USEVARIABLES =  W0EFD W0EFEX W0EFHO W0EFPR W0EFSD
              W17EFD W17EFEX W17EFHO W17EFPR W17EFSD;
GROUPING = W17ARM (0=CONTROL 1=INTERVENTION);
CATEGORICAL =  W0EFD W0EFEX W0EFHO W0EFPR W0EFSD
             W17EFD W17EFEX W17EFHO W17EFPR W17EFSD;

ANALYSIS:
ESTIMATOR = WLSMV;
PARAMETERIZATION = DELTA;

MODEL:
!Factor loadings free except first indicator
PASE7_T1 BY W0EFSD W0EFD* W0EFEX* W0EFHO* W0EFPR*;
PASE7_T2 BY W17EFSD W17EFD* W17EFEX* W17EFHO* W17EFPR*;
!Thresholds free
[W0EFSD$1*];
[W0EFSD$2*];
[W0EFSD$3*];
[W0EFD$1*];
[W0EFD$2*];
[W0EFD$3*];
[W0EFEX$1*];
[W0EFEX$2*];
[W0EFEX$3*];
[W0EFHO$1*];
[W0EFHO$2*];
[W0EFHO$3*];
[W0EFPR$1*];
[W0EFPR$2*];
[W0EFPR$3*];
[W17EFSD$1*];
[W17EFSD$2*];
[W17EFSD$3*];
[W17EFD$1*];
[W17EFD$2*];
[W17EFD$3*];
[W17EFEX$1*];
[W17EFEX$2*];
[W17EFEX$3*];
[W17EFHO$1*];
[W17EFHO$2*];
[W17EFHO$3*];
[W17EFPR$1*];
[W17EFPR$2*];
[W17EFPR$3*];
```

```
!Scale factors set to 1 in control group
{W0EFD-W0EFSD@1 W17EFD-W17EFSD@1};
!Factor variances and covariances all free
PASE7_T1* PASE7_T2*;
PASE7_T1 WITH PASE7_T2*;
!Factor means set at 0 in control group
[PASE7_T1@0 PASE7_T2@0];
!Error covariances for same indicator over time
W0EFD WITH W17EFD;
W0EFEX WITH W17EFEX;
W0EFHO WITH W17EFHO;
W0EFPR WITH W17EFPR;
W0EFSD WITH W17EFSD;

MODEL INTERVENTION:
!Factor loadings free except first indicator
PASE7_T1 BY W0EFSD@1 W0EFD* W0EFEX* W0EFHO* W0EFPR*;
PASE7_T2 BY W17EFSD@1 W17EFD* W17EFEX* W17EFHO* W17EFPR*;
!Thresholds free
[W0EFSD$1*];
[W0EFSD$2*];
[W0EFSD$3*];
[W0EFD$1*];
[W0EFD$2*];
[W0EFD$3*];
[W0EFEX$1*];
[W0EFEX$2*];
[W0EFEX$3*];
[W0EFHO$1*];
[W0EFHO$2*];
[W0EFHO$3*];
[W0EFPR$1*];
[W0EFPR$2*];
[W0EFPR$3*];
[W17EFSD$1*];
[W17EFSD$2*];
[W17EFSD$3*];
[W17EFD$1*];
[W17EFD$2*];
[W17EFD$3*];
[W17EFEX$1*];
[W17EFEX$2*];
[W17EFEX$3*];
[W17EFHO$1*];
[W17EFHO$2*];
[W17EFHO$3*];
[W17EFPR$1*];
[W17EFPR$2*];
[W17EFPR$3*];
!Scale factors set at 1 in intervention group
{W0EFD-W0EFSD@1 W17EFD-W17EFSD@1};
!Factor variances and covariances all free
PASE7_T1* PASE7_T2*;
PASE7_T1 WITH PASE7_T2*;
!Factor means fixed in intervention group
[PASE7_T1@0 PASE7_T2@0];
```

```
!Error covariances for same indicator
W0EFD WITH W17EFD;
W0EFEX WITH W17EFEX;
W0EFHO WITH W17EFHO;
W0EFPR WITH W17EFPR;
W0EFSD WITH W17EFSD;

OUTPUT:
SAMPSTAT;
STANDARDIZED (STDYX);
CINTERVAL;
RESIDUAL;
MODINDICES (4);
TECH2;

SAVEDATA:
DIFFTEST = PASE7_LI_MI_CONFIGURAL_DERIVD.DAT;
```

PASE-7 SIMULTANEOUS METRIC MODEL

```
TITLE: PASE-7 SIMULTANEOUS METRIC MODEL

DATA: FILE = PASE.MPLUS.dat;

VARIABLE:
NAMES = W0EFM W0EFTV W0EFH W0EFHM W0EFS W0EFB
       W0EFD W0EFC W0EFL W0EFEX W0EFHO W0EFPR W0EFSD
       W17ARM W17EFM W17EFTV W17EFH W17EFHM W17EFS W17EFB
       W17EFD W17EFC W17EFL W17EFEX W17EFHO W17EFPR W17EFSD;
USEVARIABLES =  W0EFD W0EFEX W0EFHO W0EFPR W0EFSD
              W17EFD W17EFEX W17EFHO W17EFPR W17EFSD;
GROUPING = W17ARM (0=CONTROL 1=INTERVENTION);
CATEGORICAL =  W0EFD W0EFEX W0EFHO W0EFPR W0EFSD
             W17EFD W17EFEX W17EFHO W17EFPR W17EFSD;

ANALYSIS:
ESTIMATOR = WLSMV;
PARAMETERIZATION = DELTA;
DIFFTEST = PASE7_LI_MI_CONFIGURAL_DERIVD.DAT;

MODEL:
!Factor loadings constrained to be equal
PASE7_T1 BY W0EFSD
          W0EFD (1)
          W0EFEX (2)
          W0EFHO (3)
          W0EFPR (4);
PASE7_T2 BY W17EFSD
          W17EFD (1)
          W17EFEX (2)
          W17EFHO (3)
          W17EFPR (4);
!Thresholds free except those stated in Mplus user guide
[W0EFSD$1] (5);
[W0EFSD$2] (6);
[W0EFSD$3*];
[W0EFD$1] (8);
[W0EFD$2*];
[W0EFD$3*];
[W0EFEX$1] (11);
[W0EFEX$2*];
[W0EFEX$3*];
[W0EFHO$1] (14);
[W0EFHO$2*];
[W0EFHO$3*];
[W0EFPR$1] (17);
[W0EFPR$2*];
[W0EFPR$3*];
[W17EFSD$1] (5);
[W17EFSD$2] (6);
[W17EFSD$3*];
[W17EFD$1] (8);
[W17EFD$2*];
[W17EFD$3*];
```

```
[W17EFEX$1] (11);
[W17EFEX$2*];
[W17EFEX$3*];
[W17EFHO$1] (14);
[W17EFHO$2*];
[W17EFHO$3*];
[W17EFPR$1] (17);
[W17EFPR$2*];
[W17EFPR$3*];
!Scale factors set at 1 in control group
{W0EFD-W0EFSD@1 W17EFD-W17EFSD@1};
!Factor variances and covariances all free
PASE7_T1* PASE7_T2*;
PASE7_T1 WITH PASE7_T2*;
!Factor means at 0 in control group
[PASE7_T1@0 PASE7_T2@0];
!Error covariances for same indicator over time - method effects
W0EFD WITH W17EFD;
W0EFEX WITH W17EFEX;
W0EFHO WITH W17EFHO;
W0EFPR WITH W17EFPR;
W0EFSD WITH W17EFSD;

MODEL INTERVENTION:
!Factor loadings constrained to be equal
PASE7_T1 BY W0EFSD@1
        W0EFD (1)
        W0EFEX (2)
        W0EFHO (3)
        W0EFPR (4);
PASE7_T2 BY W17EFSD@1
        W17EFD (1)
        W17EFEX (2)
        W17EFHO (3)
        W17EFPR (4);
!Thresholds free except those stated in Mplus user guide
!Thresholds free
[W0EFSD$1] (5);
[W0EFSD$2] (6);
[W0EFSD$3*];
[W0EFD$1] (8);
[W0EFD$2*];
[W0EFD$3*];
[W0EFEX$1] (11);
[W0EFEX$2*];
[W0EFEX$3*];
[W0EFHO$1] (14);
[W0EFHO$2*];
[W0EFHO$3*];
[W0EFPR$1] (17);
[W0EFPR$2*];
[W0EFPR$3*];
[W17EFSD$1] (5);
[W17EFSD$2] (6);
[W17EFSD$3*];
[W17EFD$1] (8);
```

```
[W17EFD$2*];
[W17EFD$3*];
[W17EFEX$1] (11);
[W17EFEX$2*];
[W17EFEX$3*];
[W17EFHO$1] (14);
[W17EFHO$2*];
[W17EFHO$3*];
[W17EFPR$1] (17);
[W17EFPR$2*];
[W17EFPR$3*];
!Scale factors free in intervention group
{W0EFD-W0EFSD W17EFD-W17EFSD};
!Factor variances and covariances all free
PASE7_T1* PASE7_T2*;
PASE7_T1 WITH PASE7_T2*;
!Factor means free in intervention group
[PASE7_T1 PASE7_T2];
!Error covariances for same indicator
W0EFD WITH W17EFD;
W0EFEX WITH W17EFEX;
W0EFHO WITH W17EFHO;
W0EFPR WITH W17EFPR;
W0EFSD WITH W17EFSD;

OUTPUT:
SAMPSTAT;
STANDARDIZED (STDYX);
CINTERVAL;
RESIDUAL;
MODINDICES (ALL);
TECH2;

SAVEDATA:
DIFFTEST = PASE7_LI_MI_METRIC_DERIVD.DAT;
```
